# Denoising spatially resolved transcriptomics with consistency of heterogeneous spatial coordinates, transcription, and morphology

**DOI:** 10.1093/bib/bbaf528

**Published:** 2025-10-04

**Authors:** Haiyue Wang, Peng Gao, Shaoqing Feng, Xiaoke Ma

**Affiliations:** School of Computer Science and Technology, Xidian University, No. 2 South Taibai Road, 710071 Xi’an, Shaanxi, China; School of Physics and Electronic Science, Shandong Normal University, No. 1 Daxue Road, 250358 Jinan, Shandong, China; Department of Hematology, The First Affiliated Hospital of Xi’an jiaotong University, No. 277 Yanta West Road, 710061 Xi’an, Shaanxi, China; Genome Institute, The First Affiliated Hospital of Xi’an jiaotong University, No. 277 Yanta West Road, 710061 Xi’an, Shaanxi, China; Department of Plastic and Reconstructive Surgery, Shanghai Ninth People’s Hospital, Shanghai Jiaotong University, No. 639 Zhizaoju Road, 200011 Shanghai, China; School of Computer Science and Technology, Xidian University, No. 2 South Taibai Road, 710071 Xi’an, Shaanxi, China

**Keywords:** spatial transcriptomics, spatial domain, feature denoising, contrastive learning

## Abstract

Spatially resolved transcriptomics (SRT) simultaneously captures spatial coordinates, pathological features, and transcriptional profiles of cells within intact tissues, offering unprecedented opportunities to explore tissue architecture. However, SRT data often suffer from substantial technical noise introduced by experimental procedures, posing challenges for downstream analyses. To overcome these challenges, we introduce a Multiview Denoising framework for Spatial Transcriptomics (MvDST), which integrates a deep autoencoder and self-supervised learning to jointly reconstruct expression profiles, denoise features, and enforce cross-view consistency, effectively reducing technical noise, and heterogeneity. As a result, MvDST reliably and accurately delineates tissue subgroups across simulated datasets under various perturbations. In real cancer datasets, it distinguishes tumor-associated domains, identifies region-specific marker genes, and reveals intra-tumoral heterogeneity. Furthermore, we validate the robustness of MvDST across multiple spatial transcriptomics platforms, including 10 $\times $ Visium, STARmap, and osmFISH. Overall, these results demonstrate that MvDST can serve as a crucial initial step for the analysis of spatially resolved transcriptomics data.

## Introduction

In multicellular organisms, cells are organized into distinct subpopulations (cell types) whose spatial distributions reflect tissue architecture and functional heterogeneity, playing critical roles in maintaining tissue homeostasis and influencing the microenvironment [1–3]. Traditional microscopy-based methods classify cell types based on morphological features, such as shape, size, color, and protein expression [4, 5]. However, these approaches are often limited by low efficiency and high time demands, owing to the intrinsic instability of cell states [6].

Single-cell RNA sequencing (scRNA-seq) measures genome-wide expression data at the level of individual cells, providing new opportunities for identifying cell types by analyzing cellular transcriptomes [7]. However, the dissociation process in scRNA-seq results in the loss of spatial coordinates of cells within the original tissues, hindering the understanding of cellular organization and function [8]. Spatially resolved transcriptomics (SRT) simultaneously obtains spatial location and transcriptional information from cells, which can be aligned with corresponding pathology images. This facilitates the study of biological mechanisms in the spatial context of cell types (termed spatial domains) [9]. According to the principles of preserving spatial information, current SRT technologies are broadly classified into two major groups: imaging-based and next-generation sequencing (NGS)-based methods. Imaging-based techniques employ probes designed for specific genes to localize mRNA transcripts, including STARmap [10], seqFISH [11], and MERFISH [12]. However, image-based approaches are criticized for their limited genome coverage, which poses an obstacle to fully understanding tissue complexity. To resolve this issue, NGS-based approaches have been developed by combining NGS and spatial barcode techniques to achieve whole-genome coverage, including 10$\times $ Visium [13], Stereo-seq [14], and Slide-seq [15]. The accumulated SRT data facilitate investigations into biological mechanisms that are inaccessible using scRNA-seq alone [8].

Although current SRT technologies have enabled significant progress in spatial gene expression profiling, the resulting data still suffer from substantial noise due to complex experimental procedures such as tissue fixation, mRNA capture, and spatial barcode assignment. These procedures frequently introduce technical artifacts, particularly dropout events, which manifest as an overrepresentation of zero values in gene expression matrices. Such artifacts are primarily attributed to suboptimal mRNA capture efficiency and the inherent limitations of sequencing depth, resulting in false negatives that obscure true biological signals. To address these issues, several denoising methods have been proposed. For example, MIST [16] identifies tissue regions by preserving molecularly similar and spatially adjacent spots, and imputes missing values by approximating a low-rank gene expression matrix using nuclear norm minimization. DenoiseST [17] leverages spatial context and gene expression profiles to mitigate dropout noise in spatial transcriptomic clustering. DIST [18] imputes gene expression profiles at unmeasured locations and enhances the signals for both observed and imputed spots using a combination of self-supervised and transfer learning strategies.

However, the aforementioned algorithms are insufficient for addressing the expression noise resulting from technical errors and biological variability in spatial transcriptomics data. For example, expression noise includes inconsistencies in RNA extraction, amplification biases, sequencing errors, and variability due to tissue heterogeneity and cellular differences. Moreover, the complexities involved in preserving spatial information further exacerbate these issues, leading to fluctuations in transcript abundance, and inaccuracies in gene expression measurements. Furthermore, these methods overlook the spatial and imaging features of the spots/beads provided by SRT, which can potentially guide and improve the correction of SRT noise with useful external information. To tackle these challenges, Sprod [19] imputes accurate SRT gene expression based on latent graph learning of matched location and imaging data. STAGATE [20] employs an attention mechanism to capture neighboring spot similarities and integrates a cell type-aware module through preclustering of gene expressions, improving spatial domain identification, and denoising while preserving spatial expression patterns. SEDR [21] learns a latent gene expression representation with spatial information using a combined masked self-supervised deep autoencoder and variational graph convolutional autoencoder for simultaneous imputation and denoising.

Despite recent advances in algorithms for mitigating noise in SRT data, several key challenges remain unresolved. First, existing algorithms treat denoising and feature learning as separate tasks, which can lead to the inadvertent suppression of biologically relevant signals during noise reduction, thereby limiting both the effectiveness of noise mitigation and the reliability of learned representations. Second, SRT data are composed of multiple complementary modalities. Current algorithms often prioritize certain modalities over others, rather than deeply integrating all modalities, which compromises the quality of latent cell representations. Therefore, developing methods to effectively learn compatible cell features is crucial for denoising SRT data. Third, the substantial heterogeneity across modalities in SRT data poses a significant challenge, as current methods learn low-dimensional cell features without adequately accounting for multimodal heterogeneity. Thus, overcoming this heterogeneity across modalities remains a critical challenge in denoising analysis.

In this work, we develop multiview denoising framework for spatial transcriptomics (MvDST) to accurately impute gene expression by utilizing the spatial coordinates of each measurement and the corresponding imaging features, which are widely available across various SRT platforms. Specifically, MvDST enhances data quality and preserves biologically relevant signals by enforcing cross-modal consistency, thereby enabling more reliable downstream analyses such as spatial domain identification and biomarker discovery. Experimental results on both simulated and real SRT datasets demonstrate that MvDST effectively mitigates noise. These findings highlight that meticulous handling of technical noise in SRT data is a critical prerequisite for the unbiased discovery of novel biological insights.

## Materials and methods

### Data preprocessing

For clarity, we utilize “spots” to denote the basic measurement units for barcode-based SRT platforms (e.g. Visium) and “cells” to denote the basic measurement units for imaging-based SRT platforms (e.g. STARmap and osmFISH). Spots (or cells) outside of the primary tissue regions are removed, and morphological features of spots are obtained using ResNet50 [22] (details on the extraction of histological image features are provided in the Supplementary Section 1.1). The raw expression profiles are normalized and log-transformed according to library size using SCANPY [23]. Genes expressed in fewer than 10 cells are filtered using Seurat [24], and expression profiles of spots are augmented with BANKSY [25].

### Mathematical model for multiview denoising framework for spatial transcriptomics

**Figure 1:**
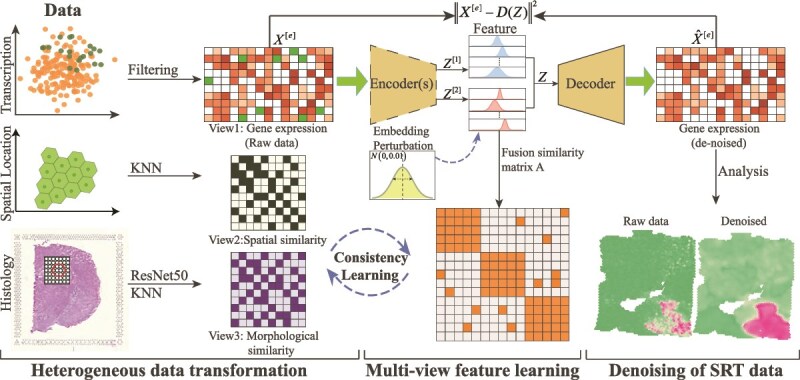
Overview of the MvDST algorithm, which consists of three components: heterogeneous data transformation, multiview feature learning, and denoising with downstream analysis. The first procedure converts SRT data into multiview representations. The multiview feature learning procedure integrates spatial, transcriptional, and morphological features through graph-based consistency learning to reduce modality heterogeneity and enhance feature quality. The final procedure reconstructs clean SRT data and evaluates denoising performance.

MvDST assumes that noise is randomly introduced into spatial coordinates, morphology, and expression profiles, and that it can be removed by learning the consistency among these three data modalities. By treating each type of data as a view, we obtain multiview data, where expression view is denoted by $Y^{[e]}$, spatial view by $Y^{[s]}$, and morphology view by $Y^{[m]}$. Thus, as depicted in Fig. 1, MvDST comprises three major components, i.e. heterogeneous data transformation, feature learning for multiview data, and denoising of SRT data.

On the heterogeneous data transformation issue, MvDST first filters high-frequency noise from the expression profile $Y^{[e]}$ with a Laplacian filter [26, 27], i.e.


(1) \begin{align*}& X^{[e]} = \phi (Y^{[e]}),\end{align*}

where $\phi $ is a filtering function [28], and $X^{[e]}$ is the filtered expression profile. MvDST then independently constructs affinity matrices $A^{[s]}$ and $A^{[m]}$ based on the spatial features $Y^{[s]}$ and morphological features $Y^{[m]}$, respectively. The morphological features $Y^{[m]}$ are extracted using ResNet50 [22]. Usually, the Pearson correlation coefficient is commonly used to construct similarity matrices for spots; however, it often results in dense matrices that hamper downstream analysis. To mitigate this issue, MvDST adopts the K-nearest neighbors (KNN) method to construct similarity matrices for spatial and morphological features.

On the feature learning for multiview data issue, MvDST first obtains transcriptional features of spots from $X^{[e]}$ using a deep autoencoder. Recently, NCE [29] has demonstrated that contrastive learning significantly enhances feature discriminability, which we adopt in our model. Specifically, MvDST employs a multilayer perceptron (MLP) to obtain initial transcriptional features as


(2) \begin{align*}& \begin{cases} Z^{[1]}=\varphi^{[1]}(X^{[e]})&\\ Z^{[2]}=\varphi^{[2]}(X^{[e]})& \end{cases}\end{align*}

where $\varphi ^{[1]}$ and $\varphi ^{[2]}$ are two different MLP. By following Haibo *et al*. [30], we add Guassian perturbation to $Z^{[2]}$, i.e. $Z^{[2]}=Z^{[2]}+\sigma $ ($\sigma \sim N(0,1)$ is Guassian distribution). Subsequently, MvDST adopts decoders $\mathcal{D}$ to reconstruct transcriptional profiles by minimizing the reconstruction loss, i.e.


(3) \begin{align*}& \mathcal{L}=\|X^{[e]}-\mathcal{D}(Z)\|^{2}\end{align*}

where $Z = \frac{1}{2}(Z^{[1]}+Z^{[2]})$. Minimizing Equation (3) enforces closeness of $Z^{[1]}$ to $Z^{[2]}$, improving consistency between them.

After obtaining the transcriptional features of spots, MvDST aims to address the consistency among transcriptional, spatial, and morphological features. The most straightforward approach is to minimize the deviation for each spot, i.e.


(4) \begin{align*}& \sum_{i}\left(\|\mathbf{z}^{[1]}_{i.}-\mathbf{y}^{[s]}_{i.}\|^{2}+\|\mathbf{z}^{[1]}_{i.}-\mathbf{y}^{[m]}_{i.}\|^{2}\right),\end{align*}

where $\mathbf{z}^{[1]}_{i.}$ is the $i$th row of $Z^{[1]}$, i.e. transcriptional feature of the $i$th spot. However, Equation (4) implies that dimensions of matrix $Z^{[1]}$, $Y^{[s]}$, and $Y^{[m]}$ are the same, which usually cannot be satisfied. To address this problem, we expect the distance among spots in all view is consistent, i.e.


(5) \begin{align*}& \sum_{ij}\left(\|\mathbf{z}^{[1]}_{i.}(\mathbf{z}^{[1]}_{j.})^{^{\prime}}-\mathbf{y}^{[s]}_{i.}(\mathbf{y}^{[s]}_{j.})^{^{\prime}}\|^{2}+\|\mathbf{z}^{[1]}_{i.}(\mathbf{z}^{[1]}_{j.})^{^{\prime}}-\mathbf{y}^{[m]}_{i.}(\mathbf{y}^{[m]}_{j.})^{^{\prime}}\|^{2}\right).\end{align*}

To further improve performance, we replace $\mathbf{z}^{[1]}_{i.}(\mathbf{z}^{[1]}_{j.})^{^{\prime}}$ with $\mathbf{z}^{[1]}_{i.}(\mathbf{z}^{[2]}_{j.})^{^{\prime}}$. To reduce computational complexity, we replace $\mathbf{y}^{[s]}_{i.}(\mathbf{y}^{[s]}_{j.})^{^{\prime}}$ and $\mathbf{y}^{[m]}_{i.}(\mathbf{y}^{[m]}_{j.})^{^{\prime}}$ with $a^{[s]}_{ij}$ and $a^{[m]}_{ij}$, respectively. Therefore, Equation (5) is rewritten as


(6) \begin{align*}& \|A-A^{[s]}\|^{2}+\|A-A^{[m]}\|^{2},\end{align*}

where $A$ is the similarity matrix of spots for transcription with elements $a_{ij}=\mathbf{z}^{[1]}_{i.}(\mathbf{z}^{[2]}_{j.})^{^{\prime}}$, and $A^{[s]}$ and $A^{[m]}$ are the affinity matrices for spatial information and morphological features, respectively.

By combining Equations (3) and (6) with a linear weighted strategy, the overall objective function of MvDST is formulated as


(7) \begin{eqnarray*} \mathcal{L} = \|X^{[e]}-\mathcal{D}(Z)\|^{2}+\alpha\|A-A^{[s]}\|^{2}+\beta \|A-A^{[m]}\|^{2}, \end{eqnarray*}

where the parameters $\alpha $ and $\beta $ control the contributions of spatial information and morphological features, respectively. Moreover, Equation (7) is minimized using the Adam optimizer [31] during training. When histology images are unavailable, $\beta $ is set to 0, and the objective function of MvDST is reformulated as


(8) \begin{eqnarray*} \mathcal{L} = \|X^{[e]}-\mathcal{D}(Z)\|^{2}+\alpha\|A-A^{[s]}\|^{2}, \end{eqnarray*}

where the parameter settings of MvDST are detailed in Supplementary Section 1.4.

### Benchmarking

To comprehensively assess the performance of MvDST, we conduct a series of experiments to illustrate its superiority over state-of-the-art algorithms. The typical denoising methods, such as Sprod [19], MIST [16], and DIST [18] are selected to fully validate the performance of denoising SRT data. The spatial domain identification algorithms, including SCANPY [23], Giotto [15], stLearn [32], SEDR [21], BayesSpace [33], SpaGCN [34], STAGATE [20], GraphST [35], DeepST [36], MFLVC [37], DCCA [38], and MUSE [39], are selected to validate quality of features learned by MvDST. All these methods are implemented with optimal parameter values (details on the experimental setup of baselines are provided in Supplementary Section 1.2).

The Adjusted Rand Index (ARI) [40] is used to evaluate the accuracy of spatial domain identification when ground truth is available. Otherwise, unsupervised clustering metrics are employed. Specifically, two metrics, Silhouette Coefficient (SC) and Davies-Bouldin Index (DBI), are selected. SC is calculated based on the mean intra-cluster distance, while DBI measures the average similarity between each cluster and its most similar cluster (sklearn: https://scikit-learn.org).

### Spatial domain identification

MvDST utilizes $K$-means [44] to obtain spatial domains with the learned features $Z$, and visualization of spatial domains is performed with uniform manifold approximation and projection (UMAP) [45].

### Identification and function enrichment analysis of differentially expressed genes

MvDST performs differential expression analysis of genes for each spatial domain using the Wilcoxon rank-sum test implemented in the SCANPY package [23]. Genes expressed in >80% of cells/spots in each domain, with $|\text{log}_{2}$(fold change)$|\geq 2$ and an adjusted FDR $\leq $ 0.05, are selected as differentially expressed genes (DEGs). Gene enrichment analysis is performed with clusterProfiler [46] (Hypergeometric test for significance).

## Results

### Overview of multiview denoising framework for spatial transcriptomics

MvDST characterizes and removes noise in SRT data by integrating histology images, spatial coordinates, and expression profiles of spots, under the assumption that noise is randomly introduced into these data and can be modeled and removed by leveraging the consistency among the heterogeneous modalities. Unlike current algorithms, MvDST transforms the denoising of SRT data into a feature learning problem within a multiview framework, where each type of heterogeneous data corresponds to a distinct view. MvDST reduces heterogeneity across views by learning consistent spot-level features, thereby effectively modeling and removing noise in SRT data.

As depicted in Fig. 1, MvDST includes three major components, i.e. heterogeneous data transformation, multiview feature learning, and denoising and downstream analysis, where the first procedure transforms spatial coordinates, expression profiles, and morphology images into multiview data by treating each of them as a separate view. In this case, integration of SRT data is converted into a multiview analysis problem, where available tools can be directly employed. The multiview feature learning procedure aims to obtain consistent features of spots from multiple views by reducing heterogeneity of data, and the last procedure restores clean expression profiles of spots and validates the performance of denoising.

On the data transformation issue, MvDST first employs a Laplacian filter [27] to remove high-frequency noise from the expression profile $Y^{[e]}$ to obtain smoothed expression data $X^{[e]}$ (Experimental Section). The spatial coordinates of spots $Y^{[s]}$ are employed to construct a spatial similarity matrix $X^{[s]}$ via K-nearest neighbors (KNN) to avoid the over-density issue associated with the Pearson coefficient strategy. For morphology images, ResNet50 [22] is adopted to obtain morphological features of spots $Y^{[m]}$, from which the morphological similarity matrix $X^{[m]}$ is constructed using KNN.

On the multiview feature learning issue, MvDST aims to learn transcriptional features of spots from the expression profile $X^{[e]}$, which are encouraged to be consistent with spatial and morphological features. Specifically, MvDST first employs two independent encoders (multilayer perceptrons, MLPs) to obtain transcriptional features of spots, where Gaussian perturbation is applied to enhance the discriminability of these features. The consistency of spatial, transcriptional, and morphological features at the spot level is achieved by narrowing the gaps among them, which is equivalent to minimizing the divergence among the spatial, transcriptional, and morphological similarity matrices. Moreover, we show that the consistency of spatial, transcriptional, and morphological features of spots can be formulated as an optimization problem (see the Experimental Section).

The final procedure obtains clean transcriptional profiles of spots using an encoder implemented as an MLP and validates the performance of denoising. Compared to existing algorithms for denoising SRT data, MvDST brings several subsequent advantages. First, MvDST transforms the integration of SRT data into a multiview data problem, enabling the direct application of machine learning tools developed for multiview data, thus providing alternatives for SRT analysis. Second, MvDST proposes a novel strategy to characterize and remove noise, serving as an effective preprocessing step for SRT data and facilitating downstream tasks such as spatial domain identification and biomarker gene discovery.

### Benchmarking multiview denoising framework for spatial transcriptomics on simulated spatially resolved data

To evaluate the effectiveness of MvDST in denoising, we first employ simulated spatially resolved data covering spatial coordinates, histology images, and transcription profiles [39] (details on the generation of simulated datasets are provided in Supplementary Section 1.3 and Table S2). There are two different measurements to validate the performance of denoising. First, we calculate the mean squared error (MSE) between the ground truth clean data $X^{[e]}$ and denoised data $\widehat{X}^{[e]}$, defined as $\frac{1}{n}\sum _{i=1}^{n}\|x^{[e]}_{i}-\widehat{x}^{[e]}_{i}\|^{2}$, where lower MSE values indicate better reconstruction, with zero representing perfect recovery. Second, since the ground truth artificial spatial domains are known, the ARI [40] is employed to evaluate the performance of different algorithms on the identification of spatial domains because the structure of domains is obvious after denoising. Three typical algorithms, including DCCA [38], MFLVC [37], and MUSE [39], are chosen as baselines. Furthermore, clustering of each single modality is also adopted to validate the performance of denoising. MIST [16], DIST [18], and Sprod [19] are excluded for a comparison on simulated datasets because the former two fail to integrate morphological images, and Sprod is incompatible with artificial morphological images (all these algorithms are selected as baselines for the biological datasets).

**Figure 2:**
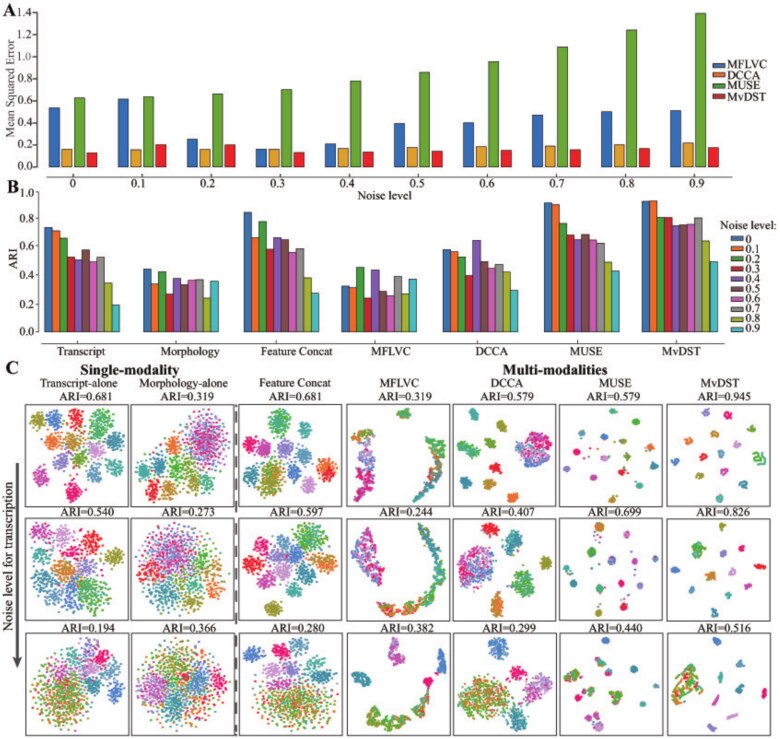
Performance of various methods in denoising the simulated data. (A) MSE between ground truth clean data and denoised data obtained by various algorithms at noise levels from 0 to 0.9. (B) ARIs for identifying ground truth clusters as the noise level in the transcriptional modality varies. (C) t-SNE visualizations of latent features from single- and multimodality approaches, with the ground truth subpopulations distinguished by different colors in the simulation.

We first assess the reconstruction performance of MvDST on simulated datasets with noise and dropout. Figure 2A illustrates how the MSE between the ground truth clean data $X^{[e]}$ and the denoised data $\widehat{X}^{[e]}$ obtained by various algorithms changes as the noise variance increases from 0 to 0.9. Although the error increases for all algorithms with greater noise variance, MvDST consistently achieves lower MSE across all levels, demonstrating superior robustness compared with the baselines. Specifically, the MSE values of MvDST on the simulated datasets are 0.129, 0.203, 0.201, 0.131, 0.137, 0.142, 0.151, 0.157, 0.167, and 0.175 as the noise variance increases from 0 to 0.9, respectively. Furthermore, we evaluate the performance of MvDST under varying transcript dropout rates while keeping the morphological and spatial modalities unchanged (Supplementary Fig. S1A). Similarly, while the MSE of all algorithms increases substantially with the dropout rate, MvDST still achieves the best performance, further confirming its effectiveness in denoising.

Moreover, to further assess the clustering performance of MvDST, we degrade the quality of the transcriptomic modality by introducing noise and dropout while keeping the spatial and morphological modalities unchanged, and evaluate the resulting spatial domain identification accuracy using ARI. Figure 2B shows the ARIs of various algorithms as the transcriptomic noise level increases. Specifically, the transcriptomic modality is corrupted with additive Gaussian noise of varying variance, while the morphological modality is perturbed with fixed-level noise, to systematically evaluate the robustness of the model under heterogeneous perturbations. Although the performance of all methods deteriorates with increasing noise variance, MvDST consistently achieves the best performance across all noise levels, demonstrating its superior robustness compared with baseline methods. Specifically, with increasing noise rates, MFLVC and DCCA underperform compared with single-modality approaches, indicating their limited ability to extract compatible features from data. In contrast, MUSE and MvDST effectively remove noise and capture discriminative features. Furthermore, as the noise variance increases from 0.1 to 0.9, the performance gap between MvDST and MUSE widens significantly, underscoring the superior precision and robustness of MvDST. Visualization of the latent spaces using t-distributed Stochastic Neighbor Embedding (t-SNE) [47] for all these approaches demonstrates that MvDST effectively models the structure of spatial domains regardless of noise level (Fig. 2C). Notably, although DCCA achieves comparable or lower reconstruction error under certain conditions, its clustering performance remains suboptimal, indicating that low reconstruction error does not necessarily imply effective discriminative feature learning. In contrast, MvDST attains an improved balance between reconstruction fidelity and feature discriminability, thereby enabling concurrent enhancements in denoising efficacy and clustering accuracy, and highlighting its methodological advantage in the analysis of spatial transcriptomics data.

Furthermore, we evaluate the clustering performance of MvDST by varying the transcript dropout rate while keeping the morphological and spatial modalities unchanged (Supplementary Fig. S2). As expected, the clustering performance of all algorithms decreases significantly as the dropout rate increases. Specifically, MUSE and MvDST exhibit notably higher robustness than other methods, indicating that these algorithms are relatively insensitive to dropout. Notably, MvDST consistently outperforms MUSE across all dropout rates, underscoring its ability to leverage high-quality features from one modality to enhance those in others. Similarly, the analysis of latent space representations across various algorithms reveals that MvDST adeptly models the spatial domain structure, regardless of the dropout rate (Supplementary Fig. S3). These results highlight the capability of MvDST to capture the intrinsic structure of heterogeneous simulated data.

Finally, to evaluate the robustness of MvDST to variations in cluster number, we vary the number of artificial spatial domains from 15 to 6 by randomly merging distinct clusters within each modality (Supplementary Fig. S4A). When the number of clusters is reduced, DCCA and MFLVC exhibit performance comparable to single-modality methods. In contrast, MUSE and MvDST significantly outperform the others, demonstrating that integrating heterogeneous data yields more accurate results than relying on single-modal inputs. Furthermore, MvDST surpasses MUSE in all these cases, suggesting that the proposed algorithm more effectively captures and models cluster perturbations in the datasets. Specifically, the ARI of MvDST is 0.823 $\pm $ 0.070 (15 clusters), 0.785 $\pm $ 0.085 (10 clusters), and 0.810 $\pm $ 0.052 (6 clusters), which represents improvements of 1.5%, 12.3%, and 3.2% over MUSE, respectively. Additionally, visualizations of the latent features learned by different methods reveal that MvDST effectively extracts discriminative and compatible features, accurately capturing the structure of spatial domains (Supplementary Fig. S4B).

There are two possible reasons that explain why the proposed algorithm outperforms existing baselines on denoising simulated datasets. First, current algorithms simply combine heterogeneous modalities via concatenation, which fails to capture the compatibility among spatial, transcriptional, and morphological features. In contrast, MvDST learns consistent representations of spots, thereby enhancing feature compatibility. Second, existing methods characterize and model noise at the individual spot level, whereas MvDST addresses this issue by considering the global structure across all spots.

### Performance of various algorithms in denoising cancer spatially resolved data

**Figure 3:**
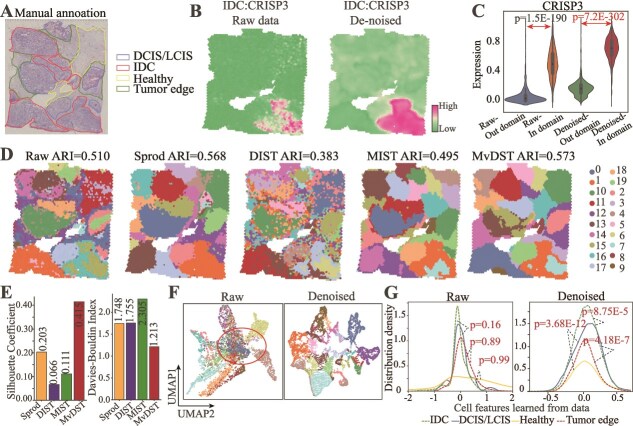
Performance of various algorithms in denoising the cancer spatially resolved data (A) Visualization of the annotation of human breast cancer data, including healthy tissue, tumor edge, IDC, and DCIS/LCIS morphotypes. (B) Visualizations of the original (left), reconstructed data (right) in breast cancer data. (C) The expression of domain-marker genes in breast cancer data (Student’s t-test for significance). (D) Visualization of spatial domains in breast cancer data identified by SCANPY based on the raw (left) and denoised (right) SRT data obtained by Sprod, DIST, MIST, and MvDST, respectively. (E) SC (left) and DBI (right) scores of various algorithms. (F) UMAP visualizationsof domains for breast cancer obtained by raw (left), and denoised data (right), respectively. (G) Distribution density of cell features for healthy tissue, tumor edge, IDC, and DCIS/LCIS domains, using original (left) and denoised (right) SRT data. The *x*-axis represents features, and the *y*-axis shows the distribution density.

Spatial transcriptomics technologies are extensively employed in disease research, prompting an exploration into the generalization capability of MvDST for elucidating tumor heterogeneity using spatially resolved cancer data. To this end, we investigate the performance of MvDST for denoising by comparing it with the typical SRT data denoising algorithms, i.e. Sprod [19], MIST [16], and DIST [18] on the publicly available human breast cancer SRT data (invasive ductal carcinoma, IDC) generated by 10$\times $ Visium, comprising 3798 spots and 36 601 genes. Figure 3A illustrates the breast cancer SRT data, manually annotated by pathologists using H&E images and spatial expression profiles of established breast cancer marker genes [21]. The dataset encompasses 20 distinct regions and is categorized into four primary morphotypes: ductal carcinoma *in situ*/lobular carcinoma *in situ* (DCIS/LCIS), IDC, tumor edge regions, and healthy tissue (Healthy).

Considering the critical role of biomarker genes in disease progression and prognosis prediction [48], we first evaluate the expression of domain-specific marker genes across various domains in both the raw and denoised breast cancer data. Figure 3B visualizes the expression of CRISP3 in both the raw and denoised data. Overall, the boundaries of expression are more distinct in the denoised data compared with the raw data, highlighting the effectiveness of the denoising process. Fortunately, this enhancement in clarity is also evident across other domains, such as APOE (Tumor Edge), CPB1 (DCIS/LCIS), and ACKR1 (Healthy) [36] (Supplementary Fig. S5A). It is evident that, due to noise in the original data, the biomarker genes do not align with the structure of the domains. However, in the denoised data, biomarker genes are accurately localized within their respective domains. Subsequently, we assess the expression levels of domain-specific biomarker genes both within and outside their corresponding domains in the denoised data. The results reveal that these biomarker genes are significantly more expressed within their designated domains than outside of them (Student’s t-test, Fig. 3C and Supplementary Fig. S5B). Even though differences in domain-specific biomarker genes are significant in the raw data, they are less pronounced compared with those observed in the denoised data. These findings indicate that MvDST effectively mitigates noise in spatially resolved transcriptomics data by enhancing the expression of domain-specific biomarkers, thereby improving the overall quality of the data.

Spatial domains are vital for analyzing the organization and functional properties of tissues [49], and numerous methods are dedicated to addressing this problem. To further assess the quality of the denoised SRT data, we selected the third-party method SCANPY [23] for spatial identification on both the raw and denoised data. Figure 3D depicts the spatial domains identified from both the raw and denoised transcript data using various algorithms, where MvDST exhibits superior performance over all baseline methods. Specifically, SCANPY, using original data, achieves an ARI of 0.516, which improves to 0.573 with MvDST denoised data, outperforming Sprod (ARI = 0.568), DIST (ARI=0.383), and MIST (ARI=0.495). After MvDST denoising, the cancer-related spatial domains detected by SCANPY are closely consistent with manual annotations, showing strong regional continuity and minimal outliers compared with the baseline. Moreover, we employ the SC and the DBI to evaluate the compactness and separation of the spatial domains. MvDST achieves a higher SC and a lower DBI compared with the baselines, indicating that the spatial domains identified by MvDST are more precise from a computational perspective (Fig. 3E).

To explore the association between cancer spatial domains and latent features before and after denoising, Fig. 3F visualizes the domains identified from the raw and denoised data using UMAP [45], highlighting the clear separation of these domains after denoising. We then investigate whether the raw and MvDST-denoised data effectively characterize tumor heterogeneity in breast cancers by distinguishing between the four major morphotypes. Figure 3G illustrates the distribution density of cell features obtained from both raw and MvDST-denoised data. Notably, denoised data effectively discriminates DCIS/LCIS, IDC, healthy, and tumor edge morphotypes. In contrast, raw data struggle to distinguish these major morphotypes, underscoring the critical role of spatial coordinates and morphological features in elucidating spatial domains within intricate tissues. Notably, the distribution density of cell features derived from the denoised data not only distinguishes these major morphotypes with high accuracy but also captures the evolutionary progression of morphotypes in breast cancer, progressing from healthy tissue to tumor periphery, and subsequently to IDC (Fig. 3G, right panel; healthy vs. tumor edge: $p = 6.12$E-9; tumor edge vs. DCIS/LCIS: $p = 4.18$E-7, Kolmogorov–Smirnov test). These findings indicate that the proposed denoising strategy effectively captures the fundamental structure of complex cancer-related domains, offering crucial insights into tumor biology.

Moreover, to assess whether the enhancement in denoising is influenced by the algorithms themselves, we visualize the spatial domains identified by different methods on both raw and denoised data using three representative approaches (SEDR [21], SpaGCN [34], and STAGATE [20]). Each row represents a clustering algorithm, while each column corresponds to the visualization of spatial domains derived from either raw or denoised data using the respective method (Supplementary Fig. S6). It is evident that all algorithms perform better on breast cancer data denoised by MvDST, whereas data denoised by baseline approaches generally leads to decreased algorithm performance, except for Sprod. Moreover, the identification performance of the data denoised by MvDST is significantly superior to that of Sprod across various algorithms. These findings further validate the superior denoising performance of MvDST compared with baseline methods.

Then, to validate the quality of features learned by MvDST, we evaluate the performance of MvDST in comparison with six state-of-the-art clustering methods for identifying spatial domains: SEDR [21], stLearn [50], SpaGCN [34], STAGATE [20], GraphST [35], and DeepST [36]. Specifically, based on the learned features $Z$, MvDST utilizes the $K$-means algorithm [44] to cluster cells/spots, where each cluster represents a distinct spatial domain. As shown in Supplementary Fig. S7, MvDST achieves an ARI of 0.661, compared to 0.510 for STAGATE, 0.526 for GraphST, 0.590 for stLearn, 0.510 for SEDR, 0.560 for SpaGCN, and 0.570 for DeepST. These results further underscore MvDST’s precision in denoising and characterizing spatial domains in cancer data.

Furthermore, to explore spatial heterogeneity within tumors, the 20 spatial domains detected by MvDST are categorized into three groups (tumor, tumor edge, and healthy) using hierarchical clustering based on Pearson correlation coefficients (Supplementary Fig. S8A). These results highlight MvDST’s ability to effectively characterize and model tumor heterogeneity. To investigate tumor heterogeneity within the tissue, DEGs across the four major morphotypes were identified, which show strong associations with breast cancer (Supplementary Fig. S8B). For instance, genes such as STRA6 and MALAT1 in the tumor edge regions correlate with variations in the abundance of tumor-associated macrophages, which are critical for patient survival due to their role in tumor angiogenesis [51, 52]. The upregulated genes are primarily involved in immune and signaling pathways, whereas the downregulated genes are associated with cell cycle processes (Supplementary Fig. S8C). Furthermore, tumor heterogeneity results in a hierarchical organization of spatial domains, with the DCIS/LCIS domain further subdivided into two sub-domains (domains 9 and 18) [21]. MvDST accurately identifies these subdomains (Supplementary Fig. S8D), exhibiting differential expression of domain-specific biomarker genes such as COX6C and CPB1. These findings illustrate that MvDST can reveal tumor heterogeneity at multiple levels, from macro-level spatial domains to micro-level features and gene expression patterns.

### Performance of various algorithms in denoising normal tissues

**Figure 4:**
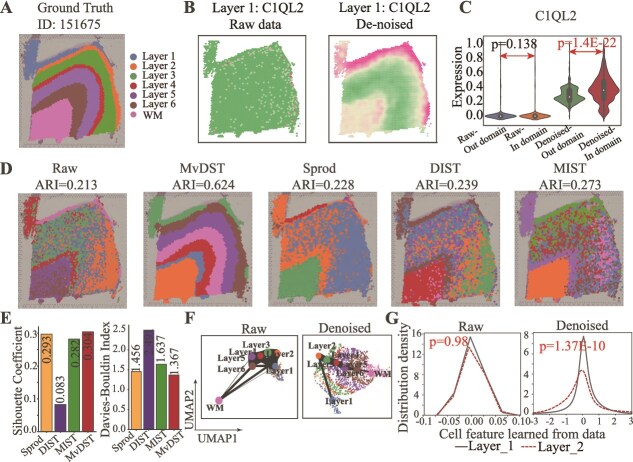
Performance of various algorithms in denoising normal tissues. (A) Ground truth segmentation of cortical layers and WM in slice 151675 of DLPFC data. (B) Visualizations of the raw data (left), reconstructed data (right) in slice 151675. (C) The expression of layer-marker genes in slice 151675 of DLPFC data (Student’s t-test for significance). (D) Visualization of spatial domains detected for denoised data by MvDST, Sprod, DIST, and MIST in slice 151675. (E) SC (left) and DBI (right) scores of various algorithms. (F) UMAP visualizations and PAGA graphs of domains for slide 151675 obtained by raw (left), and denoised data (right), respectively. (G) Distribution density of cell features for raw (left) and denoised data (right) for Layer 1 and Layer 2.

We next investigate the performance of MvDST for denoising using spatially resolved datasets from normal tissues, i.e. the LIBD human dorsolateral prefrontal cortex (DLPFC) dataset [41]. The DLPFC dataset comprises 12 tissue slices from three human brains. Each slice has been manually annotated into either six layers of the DLPFC (Layers 1 through 6) or four layers (Layers 3 through 6), as well as white matter (WM), based on morphological features and gene markers (Fig. 4A shows slice 151675 of the DLPFC data). We investigate the performance of MvDST for denoising by comparing it with three typical SRT data denoising algorithms and nine state-of-the-art clustering methods for identifying spatial domains. The denoising methods include Sprod [19], MIST [16], and DIST [18]. The clustering methods include the nonspatial method SCANPY [23] and the spatial methods Giotto [15], SEDR [21], stLearn [50], SpaGCN [34], BayesSpace [33], STAGATE [20], GraphST [35], and DeepST [36]. The ARI was employed to assess the performance of these algorithms, given the availability of ground truth spatial domain labels.

We begin by evaluating the expression of layer-specific marker genes in slice 151675, comparing the data from the raw and denoised datasets for each cortical layer (Fig. 4B and Supplementary Fig. S9A and B). Specifically, Fig. 4B visualizes the expression of C1QL2 (Layer 1) in the original (left) and denoised data (right), demonstrating improved clarity and more distinct and continuous boundaries between layers. Subsequently, we also evaluate the expression of layer-specific biomarker genes both within their corresponding layers and in regions outside these layers in the denoised dataset. Notably, in the raw data, the distinction between gene expression within the corresponding layer and in regions outside of it is not statistically significant (Fig. 4C, left, Student’s t-test, $p=0.138$) but becomes significant in the denoised data (Fig. 4C, right, Student’s t-test, $p=1.4$E-22). These findings indicate that MvDST effectively reduces noise in normal SRT data, leading to improved expression levels of layer-specific marker genes. In summary, MvDST leverages multiview feature denoising and consistency learning to accurately characterize and eliminate noise, positioning itself as a crucial preprocessing tool for SRT data.

Then, we examine the effectiveness of denoised data for spatial domain identification in normal tissues. Figure 4D illustrates the spatial domains detected using the raw and restored transcriptomic data with various algorithms, where MvDST exhibits superior performance over all baseline methods. It is worth noting that in normal tissues, the performance of Sprod and DIST is inferior to their performance in cancer tissues. Specifically, SCANPY achieves an ARI of 0.213 using original data, which improves to 0.624 with MvDST-denoised data, outperforming Sprod-denoised (ARI = 0.228), DIST-denoised (ARI = 0.239), and MIST-denoised (ARI = 0.273). The domains in the raw data are noisy with indistinct boundaries, whereas in the MvDST-denoised data, these domains are precisely and clearly detected. Notably, Layers 1 and 2 are clearly distinguished in the denoised data, indicating that MvDST proficiently uncovers the inherent structure of spatially resolved data by mitigating noise. Moreover, Fig. 4E shows the SC and DBI scores of various algorithms, indicating that the spatial domains identified by MvDST are more compact and better separated than those of other methods. For example, the SC of MvDST is 0.304, while that of the best-performing baseline, Sprod, is 0.293. Similarly, the DBI of MvDST is 1.367, compared with 1.456 for Sprod.

Moreover, understanding the trajectory of spatial domains is essential for uncovering biological evolution mechanisms [53]. We employ PAGA [54] to infer relationships among spatial domains detected by the original and denoised data. The MvDST-denoised data accurately detects the organization of cortical layers from Layer 1 to Layer 6 and WM, while datasets without denoising mistakenly connect diverse spatial domains (Fig. 4F). These findings illustrate that MvDST precisely identifies the underlying structure and evolutionary relationships of spatial domains. Furthermore, we compared the distribution of cell features obtained by SCANPY for Layers 1 and 2 from both the raw and denoised transcripts. In the raw data, the distinction between these two spatial domains is not significant (Fig. 4G, left), but it becomes significant in the denoised data (Fig. 4G, right). Specifically, the standard deviation of cell features in the raw data is 0.14 for Layer 1 and 0.29 for Layer 2 ($p=0.98$, Kolmogorov–Smirnov test), whereas in the denoised data, it is 1.79 and 0.21, respectively ($p=1.37$E-10, Kolmogorov–Smirnov test). These findings indicate that MvDST effectively denoises spatially resolved data by leveraging feature learning and multiview relationships among spots, thereby improving the discriminative capacity of cell features.

To ascertain whether the enhancement in denoising is affected by the methods or by specific slices of the DLPFC data, we apply all baseline algorithms for spatial domain detection to both the raw and restored DLPFC data. The distributions of ARIs for these algorithms on the DLPFC data are shown in Supplementary Fig. S9C. Remarkably, all algorithms demonstrated significantly better performance on the restored data compared with the raw data, indicating that the performance enhancement is not influenced by the algorithms or specific slices. Additionally, SCANPY, SpaGCN, and stLearn show significant improvements in spatial domain identification, and other baselines also exhibit enhanced performance with the restored data. For instance, the ARI for SCANPY shows a significant increase from 0.207 $\pm $ 0.049 to 0.464 $\pm $ 0.085 ($p=1.0$E-8, Student’s t-test). In contrast, the ARI for stLearn improves from 0.272 $\pm $ 0.059 to 0.321 $\pm $ 0.057 ($p=0.42$, Student’s t-test). Despite the lack of significant improvement for SEDR and STAGATE, the denoising process results in a 2.1% and 1.9% enhancement, respectively. The suboptimal performance of STAGATE can be attributed to its reliance on an auto-encoder strategy for denoising, which results in redundant denoising steps that diminish its efficacy. These findings underscore the potential of multiview fusion for effectively understanding and depicting noise in SRT data. Furthermore, they highlight the utility of MvDST as a robust preprocessing tool for downstream analysis.

Finally, to validate the quality of features learned by MvDST, we assess the performance of MvDST combined with $K$-means [44] in comparison with nine state-of-the-art clustering methods for identifying spatial domains. MvDST also achieves significant superiority over baselines in identifying spatial domains (Supplementary Figs S10–S12). In detail, ARI of MvDST is 0.644 $\pm $ 0.084, whereas that of GraphST is 0.527 $\pm $ 0.083, STAGATE is 0.492 $\pm $ 0.042, SpaGCN is 0.451 $\pm $ 0.057, DeepST is 0.515 $\pm $ 0.033, SEDR is 0.385 $\pm $ 0.062, stLearn is 0.272 $\pm $ 0.059, Giotto is 0.243 $\pm $ 0.078, SCANPY is 0.207 $\pm $ 0.049, and BayesSpace is 0.459 $\pm $ 0.104, based on the analysis of 12 tissue slices. stLearn, SCANPY, and Giotto are deemed inferior to other algorithms due to their reliance on a single modality or limited ability to integrate multiple modalities. In contrast, MvDST exhibits higher robustness compared with these methods, demonstrating superior performance across all datasets. These results highlight the capability of MvDST to effectively learn discriminative cell features for all slices. We further perform a comprehensive parameter analysis of MvDST on the DLPFC slice, demonstrating its robustness (Supplementary Section 1.4).

### Multiview denoising framework for spatial transcriptomics is applicable for spatially resolved data with various platforms

**Figure 5:**
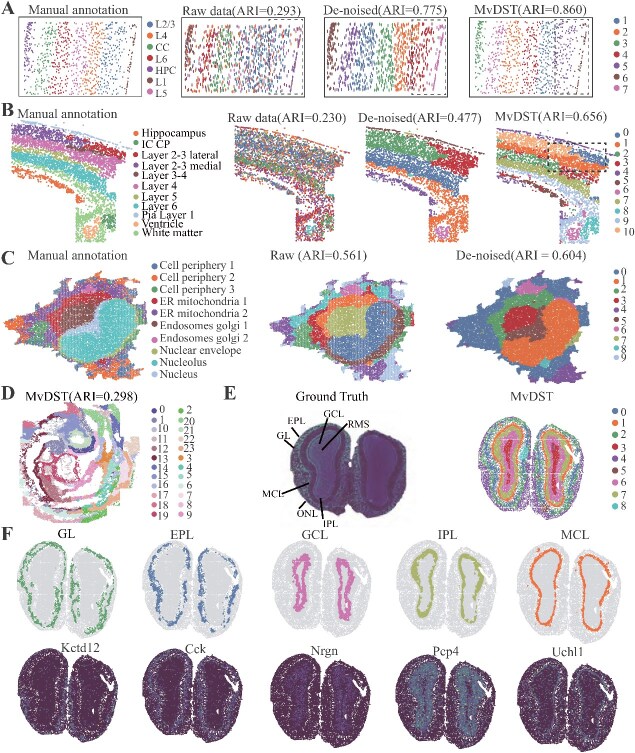
MvDST is applicable for spatially resolved data with various platforms. (A) Ground truth of regional annotation of starMap SRT data (left), and spatial domains detected by SCANPY from the raw data, SCANPY from MvDST-denoised data, and MvDST combined with $K$-means clustering. (B) Manual annotation of osmFISH data (left), and spatial domains identified by SCANPY from the raw data, SCANPY from MvDST-denoised data, and MvDST combined with $K$-means clustering. (C) Visualization of subcellular molecular profiles using 4i (iterative indirect immunofluorescence imaging), plotted in spatial coordinates (left, 17 253 observations/pixels and 43-plex proteins, annotated 10-cell states), and spatial domain identification using raw data (middle) and MvDST-denoised data (right) were plotted ER, endoplasmic reticulum. (D) Spatial domains identified by MvDST using the SeqFISH data with a fixed number of clusters ($k=24$) as a clustering parameter. (E) Visualization of SRT data generated with the Stereo-seq platform (left), and spatial domains identified by MvDST (right). MCL, mitral cell layer; IPL, internal plexiform layer; GCL, granule cell layer; RMS, rostral migratory stream. (F) Visualization of spatial domains identified by MvDST for each tissue and the corresponding marker gene expressions, where each column denotes a tissue.

With the exception of the 10$\times $ Visium platforms, we further showcase MvDST using the mouse primary visual cortex 1020 gene dataset obtained via STARmap technology (Fig. 5A). RNA clusters per cell were derived from ClusterMap predictions, and seven distinct layers (or regions) were annotated based on raw fluorescence data (Fig. 5A, left panel).

Figure 5A right panel shows the spatial domains identified using the raw and restored data with SCANPY, where the restored data exhibits superior performance compared with the raw data. Specifically, SCANPY, using the raw data, achieves an ARI of 0.293, which improves to 0.775 with MvDST-denoised data. Additionally, we assess the performance of MvDST combined with K-means in comparison with other clustering methods for identifying spatial domains. In detail, the ARI of MvDST is 0.860, whereas that of GraphST is 0.473, STAGATE is 0.528, and DeepST is 0.776 (Supplementary Fig. S13A). Compared with DeepST, MvDST exhibits superior performance in spatial domain identification due to its effective denoising strategy.

We further explore the applicability of the MvDST model with four SRT datasets where morphological information is absent: mouse brain cortex obtained by osmFISH, imaging-based molecular data 4i [36], mouse olfactory bulb tissue obtained by Stereo-seq [21], and mouse embryogenesis data obtained by SeqFISH [55]. We initially assess the performance of MvDST using nonlattice-shaped SRT data generated by osmFISH, where spatial domains are distinguished by different colors (Fig. 5B). SCANPY, using raw data, achieves an ARI of 0.230, which improves to 0.477 with MvDST-denoised data. In comparison to state-of-the-art algorithms for spatial domain identification, MvDST achieves competitive results (Fig. 5B and Supplementary Fig. S13B; MvDST: ARI = 0.656), underscoring that denoising in the transcriptional modality effectively enhances the discriminative power of the learned latent features.

We then apply MvDST to 4i (iterative indirect immunofluorescence imaging) data, which measure 40 protein reads in high-throughput biological samples across scales from millimeters to nanometers (270 000 observations/pixels). Specifically, we utilize partial molecular data for spatial domain identification (Supplementary Fig. S13C, 17 253 observations). MvDST with denoising reveals a more detailed subcellular distribution within local areas, encompassing various compartments, organelles, and cellular structures within each cell (Fig. 5C, raw data ARI = 0.561; MvDST-denoised data ARI = 0.604).

We also apply MvDST to mouse embryo tissue to assess its broad applicability. In the original study [43], the embryo tissue section was annotated with 24 tissue structures (embryo 1, Supplementary Fig. S13D), which were used for comparison with domain segmentations generated by MvDST. Overall, MvDST separates the outer layers of the organ, namely the cardiomyocytes, neural crest, and gut tube (Fig. 5D). Finally, we apply MvDST to coronal mouse olfactory bulb tissue acquired with Stereo-seq. We first annotate the coronal mouse olfactory bulb’s laminar structure using the DAPI-stained image, including the olfactory nerve layer (ONL), mitral cell layer, glomerular layer (GL), internal plexiform layer, external plexiform layer (EPL), granule cell layer, and rostral migratory stream. Overall, MvDST separates the outer layers of the organ, namely the ONL, GL, and EPL (Fig. 5E).

Finally, we utilize marker genes specific to each anatomical region to assess the validity of the domains detected by MvDST. We observe strong agreement between the MvDST-identified domains and the known marker genes. Specifically, markers such as Pcp4 and Uchl1 show high expression levels consistent with neighboring regions (Fig. 5F). This outcome is expected, as cell types often overlap across various internal organ structures, and markers are similarly shared among related cell types. Overall, MvDST adeptly utilizes the entire transcriptome and spatial information to distinguish the relevant anatomical regions. These results highlight the effectiveness of MvDST in feature denoising and consistency learning, thereby enhancing the identification and characterization of spatial domains.

## Discussion

Advances in NGS technologies enable gene transcription measurements at the cellular level while preserving spatial context, offering significant insights into biological mechanisms. Analyzing spatial transcriptomics data deepens the understanding of tissue structure and function, revealing how cell populations contribute to various regions of an organism. This approach provides a notable advantage over traditional bulk sequencing methods, which fail to capture spatial variations within tissues. However, the analysis of spatial transcriptomics data presents considerable challenges due to its extreme sparsity, heterogeneity, and noise, which complicate the development of effective algorithms.

In this study, we propose MvDST, a flexible multiview deep autoencoder model that can effectively and efficiently integrate histology images, spatial coordinates, and transcriptional information in SRT data for data denoising. Comprehensive experiments demonstrate that denoising through MvDST is a crucial initial step in enhancing spatially resolved transcriptomics technologies, with its advantages highlighted in several aspects. First, MvDST constructs a multiview representation by combining diverse data types, enhancing feature compatibility through multiview feature learning and consistency learning. This modeling strategy can tolerate high-dimensional data noise, preserve critical spatial and image feature relationships, and integrate histology images, spatial coordinates, and gene expression data within a unified framework (Fig. 2). Second, MvDST precisely removes noise from cancer spatial domains and accurately identifies spatial domains, providing valuable insights for disease research, biomarker discovery, and understanding disease mechanisms (Fig. 3). Third, MvDST contributes to mapping tissue architecture and facilitates the discovery of relevant gene markers, offering an efficient and effective strategy for understanding complex biological systems within spatial environments (Fig. 4). Finally, MvDST demonstrates strong scalability and can be effectively applied to spatial transcriptomics data from various platforms (Fig. 5).

MvDST integrates spatial, transcriptional, and morphological features through graph-based consistency learning, aiming to reduce modality heterogeneity and enhance feature quality, thereby enabling a more effective characterization of tissue structure than single-modality methods. We demonstrate that MvDST accurately identifies spatial domains in both normal and tumor tissues across different species and platforms. Furthermore, we conduct comprehensive ablation studies on various datasets (Supplementary Section 1.5) to evaluate the contributions of each component, demonstrating that all components are indispensable and further validating the effectiveness of MvDST.

Pathology plays a critical role in cancer diagnosis and therapy. Therefore, enhancing the capability of MvDST to capture pathological features is highly significant for clinical applications. Recently, two biological image-specific models, UNI [56] and Prov-GigaPath [57], have been proposed as foundation models for computational pathology. We explore the potential of incorporating these models into MvDST by replacing ResNet50 with either UNI or Prov-GigaPath. Supplementary Fig. S18 presents the performance of these MvDST variants on the human breast cancer and DLPFC datasets.

As illustrated in Supplementary Fig. S18A, biological image-specific models improve performance on H&E-stained cancer images. Specifically, the ARIs for UNI and Prov-GigaPath are 0.449 and 0.430, respectively, compared to 0.300 for ResNet50. Incorporating these models into MvDST leads to an improvement in performance, with the ARI increasing from 0.573 to 0.664. However, these models perform suboptimally on normal tissues (Supplementary Fig. S18B and C), where the ARIs for UNI and Prov-GigaPath are 0.269 and 0.243, respectively, slightly lower than the 0.272 achieved by ResNet50. Correspondingly, the ARI of MvDST decreases slightly from 0.624 to 0.620. The evaluation across all 12 slices of the DLPFC dataset (Supplementary Fig. S18C) confirms the consistency of these findings. This discrepancy arises from the substantial differences between morphological images of normal and tumor tissues, with biological image-specific models demonstrating superior effectiveness in capturing tumor heterogeneity. Overall, these results highlight the flexibility of MvDST in integrating diverse morphological feature extractors and further underscore its potential for clinical applications.

An important limitation of our work is that we only utilize the similarity relationships of image data without incorporating specific image features. Future studies could improve results by integrating detailed image features. Despite recent advances in SRT technologies, data quality remains a significant challenge, particularly due to multicell mixtures within spots. To better address this issue, we plan to integrate high-resolution cell segmentation or spatial deconvolution methods to disentangle cellular composition and enhance interpretability. Moreover, the integration of confidence intervals, posterior variance, or bootstrapped estimates would further improve the interpretability of the model and the robustness of downstream results. Although the method demonstrates improvement, clustering metrics (e.g. Silhouette Score) remain modest, reflecting the complexity of spatial transcriptomics data and motivating further enhancement of denoising and discriminative feature learning for more robust, biologically meaningful clustering. We believe that MvDST, explicitly developed for accurate SRT gene expression denoising, will be a crucial first step in leveraging SRT technologies for biomedical breakthroughs and advancements.

**Key Points**

- We propose a novel framework that leverages cross-modal consistency to mitigate noise effects in spatially resolved transcriptomics (SRT) data, thereby improving data quality and enabling more accurate downstream analyses, such as spatial domain identification and biomarker discovery.
- We introduce a multiview feature consistency strategy that aligns spatial, transcriptional, and morphological information at the spot level by jointly optimizing their similarity structures through dual Gaussian-perturbed encoders, enhancing both robustness and feature discriminability.
- The experimental findings on both simulated and SRT data indicate that multiview denoising framework for spatial transcriptomics not only effectively mitigates noise but also enhances the elucidation and understanding of spatial tissue heterogeneity.

## Supplementary Material

Supplementary_bbaf528

## Data Availability

The code for the MvDST algorithm and a detailed tutorial are available at https://github.com/zilanjiuwan/MvDST. All datasets utilized in this paper are publicly available for download (Supplementary Table S1). (i) Human DLPFCs are accessible within the SpatialLIBD [41] at http://spatial.libd.org/spatialLIBD; (ii) human breast cancer is collected from the 10 $\times $ Genomics website at https://support.10xgenomics.com/spatial-gene-expression/datasets; (iii) STARmap mouse cortex dataset: raw data were downloaded from the project page http://clarityresourcecenter.org/. Transcript profiles and cell segmentation masks were extracted from data using the Python pipeline provided by the authors at https://github.com/weallen/STARmap; (iv) the mouse brain cortex osmFISH data [42] is accessible at http://linnarssonlab.org/osmFISH; (v) the processed Stereo-seq data from mouse olfactory bulb tissue is accessible on https://github.com/JinmiaoChenLab/SEDRanalyses; (vi) 4i dataset is accessible at https://github.com/scverse/squidpy; and (vii) the processed SeqFISH mouse embryogenesis dataset [43] with segmentation information and associate metadata are available at https://crukci.shinyapps.io/SpatialMouseAtlas/.
