# Supplementary material for: Denoising spatially resolved transcriptomics with consistency of heterogeneous spatial coordinates, transcription, and morphology
Source: Brief Bioinform. 2025 Oct 4;26(5):bbaf528. doi: 10.1093/bib/bbaf528 (PMC12496014; doi:10.1093/bib/bbaf528)
Supplement: Supplementary_bbaf528 [file supplementary_bbaf528.pdf]

Supplementary Information for

Denoising Spatially Resolved Transcriptomics with Consistency of Heterogeneous Spatial Coordinates, Transcription and Morphology

Haiyue Wang[1,2], Peng Gao[3,4,*], Shaoqing Feng[5,*], and Xiaoke Ma[1,*]

[1]School of Computer Science and Technology, Xidian University, No.2 South Taibai Road, Xi'an Shaanxi, 710071, China,

[2]School of Physics and Electronic Science, Shandong Normal University, No.1 Daxue Road, Changqing District, Jinan, Shandong, 250358, China,

[3]Department of Hematology, The First Affiliated Hospital of Xi'an jiaotong University, No. 277 Yanta West Road, 710061, Xi'an, Shaanxi, China,

[4]Genome Institute, The First Affiliated Hospital of Xi'an jiaotong University, No. 277 Yanta West Road, 710061, Xi'an, Shaanxi, China,

[5]Department of Plastic and Reconstructive Surgery, Shanghai Ninth People's Hospital, Shanghai Jiaotong University, No. 639 Zhizaoju Road, 200011, Shanghai, China.

[*]Corresponding authors. xkma@xidian.edu.cn, fengsq112046@sh9hospital.org.cn, gaopeng@xjtu.edu.cn.

Keywords: Spatial Transcriptomics, Spatial Domain, Feature Denoising, Contrastive Learning

Supplementary Notes

1.1 Extraction of Histological Image Features

For SRT data with morphological information, we first segmented histological images (H&E-stained tiles) based on the coordinates of each spot to obtain corresponding image patches, resulting in a number of patches equal to the number of spots or cells. Specifically, for all 12 slices in the DLPFC dataset and the human breast cancer dataset generated by the Visium platform, the corresponding H&E-stained images were segmented into patches, each sized 112 × 112 pixels. We then applied the torchvision.transforms function to transform and augment these image patches, including normalization, rotation, and sharpness adjustment. High-level features for each spot patch were extracted using a pretrained convolutional neural network model (optional; ResNet50 by default), which converts each image patch into a 2048-dimensional latent vector. To better represent spot morphology, principal component analysis (PCA) was performed to extract the first 50 principal components as latent features. Notably, the extraction of morphological features excludes spots (or cells) located outside the primary tissue regions.

1.2 Experimental Setup of Baselines

1) **Sprod**: We adapted the Sprod workflow described in their online tutorial (https://github.com/yunguan-wang/SPROD). We conducted grid searches over the recommended parameter ranges, including R (0.04–0.24, step size = 0.01), K (3–10, step size = 1), U (250, 500 and 1,000), the Lambda (0.1–1, step size = 0.1, plus 5 and 20), and L_E (0.3125, 0.625, 1.25 and 2.5).
2) **MIST**: We adapted the MIST workflow described in their online tutorial (https://github.com/linhuawang/MIST.git). We performed grid searches over the recommended parameter ranges, including threshold value (0.1-0.9, step size = 0.1), and sigma=0.1.
3) **DIST**: We adapted the DIST workflow described in their online code(https://github.com/zhaoyp1997/DIST). We ran the method with the recommended parameters, including K = 2 and L = 5.
4) **SCANPY**: Raw gene expression was preprocessed to select the top 3,000 HVGs and log-normalized. We then ran PCA to extract the top 30 principal components (PCs) and then the nearest neighbor network was constructed using the scanpy.pp.neighbor() function with default parameters. Finally, SCANPY obtains the clustering assignments using the scanpy.tl.louvain() function. For the DLPFC dataset, the resolution parameter was tuned manually to ensure the number of clusters matches the ground truth.
5) **Gitto**: We adapted the Giotto workflow described in their online tutorial (http://spatialgiotto.rc.fas.harvard.edu/giotto.visium.brain.html). Specifically, the expression data was normalized by applying the scalefactor parameter set to 6000. The spatial network was first constructed using the createSpatialNetwork() function with k=5 and maximum_distance_knn=1000. Then, the spatial domains were identified using the doHMRF() function with the parameter betas set to c(0, 10, 20).
6) **stLearn**: We adapted the stLearn workflow described in their online

tutorial(https://stlearn.readthedocs.io/en/latest/stSME_clustering.html#Human-Brain-dorsolateral-prefrontal-cortex-(DLPFC). The stLearn.SME.SME_normalized() function was performed on the raw counts of all genes with parameters use_data=”raw” and weights=” physical_distance”. Then the first 30 PCs of the SME normalized matrix were used for further clustering and visualization.

7) **SEDR**: We ran SEDR for all experiments with its recommended parameters in the online tutorial (https://github.com/JinmiaoChenLab/SEDR/). Specifically, the parameter k was set to 10, and the number of epochs was set to 200.
8) **BayesSpace**: BayesSpace was applied to the DLPFC datasets as described in their online tutorial (https://github.com/edward130603/BayesSpace).The input is the top 15 PCs of the log-normalized expression of the top 2,000 HVGs. The nrep parameter was set to 50,000 and the gamma parameter was set to 3. We only ran it for 10 × Visium data because its package did not support the calculation of Stereo-seq data directly.
9) **SpaGCN**: SpaGCN was applied to the DLPFC datasets as described in their online tutorial (https://github.com/jianhuupenn/SpaGCN/blob/master/tutorial/tutorial.ipynb). We ran these methods with the recommended parameters, including s=1, b=49, p=0.5, n_clusters=7 (depending on the number of spatial domains) and max_epochs=200, and set the number of clusters to match the ground truth.
10) **STAGATE**: We ran STAGATE for all experiments using the recommended parameters provided in the official tutorial (https://github.com/QIFEIDKN/STAGATE_pyG).
11) **GraphST**: We ran GraphST for all experiments using the recommended parameters provided in the official tutorial (https://github.com/JinmiaoChenLab/GraphST).
12) **DeepST**: We ran DeepST for all experiments with its recommended parameters in the online tutorial (https://github.com/JiangBioLab/DeepST). Specifically, pca_n_comps=100, eval_cluster_n was determined based on the ground truth, distType="KDTree", pre_epochs=1000, adjacent_weight=0.3, platform="Visium", k=12, weights= "weights_matrix_all", Conv_type= "GCNConv", pretrain = False, dim_reduction = True, priori = True, linear_encoder_hidden= [64,16] and conv_hidden=[64,16].

1.3 Simulated Dataset

By following MUSE[1] and MuCST[2], we generate simulated transcriptional and morphological data by assigning similar spatial information to cells with the same labels. Specifically, we employ the scRNA-seq simulation frameworks of SIMILR[3] and scScop[4] to generate transcriptional and morphological information, introducing varying degrees of noise and dropout rates into the simulated dataset. Then, we generate simulated labels $l \in \{1,...,L\}^n$ for n cells and l possible cluster assignments, assigning similar spatial coordinates to simulated cells with the same cluster labels to ensure spatial patterns in the simulated dataset. All simulation parameters used in experiments are summarized in Table S2.

1.4 Parameter Setting

Two parameters are involved in MvDST, i.e., α and β, where α determines the importance of the spatial information, and β controls the importance of morphology feature. To investigate parameter effects, we depict how ARI of MvDST changes by varying values of parameters on different datasets, as shown in Supplementary Fig. S14, where panel **A** and **B** are for the simulated dataset, **C** for DLPFC dataset, and **D** for the human breast cancer dataset, respectively.

To fully exploit parameter effects, two types of simulated datasets are generated, i.e., balanced and unbalanced simulated dataset, where morphology and spatial are equal important in the first type of dataset, and morphology dominates in the unbalanced simulated dataset. Supplementary Fig.

S14**A** shows that MvDST achieves the best performance on the balanced simulated dataset if $\alpha \in [0.01,100]$ and $\beta \in [0.1,10]$. These results demonstrate that MvDST is quite stable when morphology and spatial modalities are equally important. Moreover, morphology and spatial information are equally important in the human breast cancer dataset (Supplementary Fig. S14**D**), where the trend is consistent. However, Supplementary Fig. S14**B** shows that MvDST achieves the best performance on the unbalanced simulated dataset if $\alpha \in [0.0001, 0.1]$ and $\beta \in [0.1,100]$.

Supplementary Fig. S14**C** shows that MvDST achieves the best performance on the DLPFC dataset if $\alpha \in [0.1,10]$ and $\beta \in [0.0001,1]$. Since the DLPFC dataset is dominated by transcriptomics and spatial, a small β reduces the importance of morphology, thereby enhancing the performance of MvDST.

Thus, considering the different relative importance of transcript and morphology modality in different real spatial omics dataset, we recommend different parameter ranges depending on the dominant modality in the dataset:

- **Spatial-dominant dataset**: we recommend small contribution of morphology, i.e., $\alpha \in [0.1,10]$ and $\beta \in [0.0001,1]$.
- **Spatial and morphology equal important dataset**: we balance morphology and transcriptomics with $\alpha \in [0.01,100]$ and $\beta \in [0.1,10]$.
- **Morphology-dominant dataset**: we recommend large value of morphology, i.e., $\alpha \in [0.0001, 0.1]$ and $\beta \in [0.1,100]$.

1.5 Ablation Study of MvDST

MvDST is based on the assumption that noise in SRT data can be effectively modeled and removed by leveraging the consistency across multiple heterogeneous modalities, including gene expression, spatial location, and histological morphology. Morphological features or spatial locations provide essential structural and contextual information regarding tissue architecture, which is often not fully captured by gene expression alone.

Thus, to evaluate contribution of spatial and morphological components, we conduct an ablation study for MvDST as well as three variants are generated, i.e., spatial input is removed (alpha=0), morphology is removed (beta=0), and both are removed (alpha=beta=0).

Performance of MvDST and its variants on different datasets is shown in Supplementary Fig. S15, where panel **A** is for human breast cancer dataset, and **B** for the DLPFC dataset. It is easy to assert that MvDST obviously outperforms MvDST (alpha=0), MvDST (beta=0), and MvDST (alpha=beta=0). For example, ARI of MvCST for slice 151675 is 0.624, whereas that of MvDST (alpha =0), MvDST (beta=0), and MvDST (alpha=beta=0) are 0.579, 0.607 and 0.529, respectively (Supplementary Fig. S15**B1**). Analogously, ARI of MvDST for all 12 slices of DLPFC dataset is 0.644 ± 0.083, whereas that of MvDST (alpha =0), MvDST (beta=0), and MvDST (alpha=beta=0) are 0.570 ± 0.051, 0.598 ± 0.078, and 0.522 ± 0.056, respectively (Supplementary Fig. S15**B2**). These findings clearly demonstrate that incorporating both spatial and morphological components enhance feature representation and facilitates effective denoising in SRT data.

To validate the contribution of BANKSY to the proposed method, we compared three approaches: a variant of MvDST without BANKSY (denoted as MvDST (without BANKSY)), MvDST, and BANKSY alone. These methods were applied to different datasets, and the spatial domains identified by each method are visualized in Supplementary Fig. S16, where panel A corresponds to the breast cancer dataset, and panel B to the slice 151675 of DLPFC dataset. It is clear that MvDST significantly outperforms both MvDST (without BANKSY) and BANKSY across different datasets. For example, the ARI of MvDST on the human breast cancer dataset is

0.661, whereas MvDST (without BANKSY) and BANKSY achieve 0.624 and 0.572, respectively (Supplementary Fig. S16A). Similarly, for slice 151675 of the DLPFC dataset, the ARI of MvDST is 0.656, compared to 0.604 for MvDST (without BANKSY) and 0.558 for BANKSY (Supplementary Fig. S16B). These results demonstrate that data augmentation improves the performance of MvDST and is a critical component of the method. Moreover, the quality of features learned by MvDST dominates over all other components.

To evaluate the contribution of perturbing the latent representation *Z*, we conduct an ablation study on MvDST by comparing the variant without perturbation, i.e., MvDST (without perturbation), and MvDST. Performance of MvDST and its variants on different datasets is shown in Supplementary Fig. S17, where panel **A** is for human breast cancer dataset, and **B** for the slice 151675 of DLPFC dataset. It is easy to assert that MvDST obviously outperforms MvDST (without perturbation). For example, ARI of MvCST for slice 151675 is 0.656, whereas that of MvDST (without perturbation) is 0.616 (Supplementary Fig. S17**B**). The tendency in the breast cancer is consistent with DLPFC dataset. These results demonstrate that perturbing the latent representation contributes to greater robustness and more accurate spatial domain identification.

1.6 Implementation Details and Training Setup

Specifically, the model consists of two parallel encoding layers (layers1 and layers2) and one shared decoding layer (layers_decoder). The encoder projects the input features from the original dimension d_0 to a latent space of dimension d_1, followed by normalization. The decoder then reconstructs the input from the averaged latent representation. The layer dimensions are defined as $[d_0, d_1]$, where d_0 corresponds to the original feature dimension of the input dataset, and d_1 is a hidden dimension. In our experiments, we set d_1 to 500. Moreover, all network weights are initialized using the Xavier uniform initialization strategy to ensure stable gradient flow during training. Our network is trained for 400 epochs until convergence by minimizing the loss in Eq. (7) with the Adam optimizer. After optimization, we directly perform the K-means algorithm on the clustering-oriented node embeddings *Z*.

1.7 Runtime Scalability of MvDST

On the $10\times$Genomics data, we independently execute each algorithm for each slide of the DLPFC dataset, where the distributions of running time and space of various algorithms are summarized in Supplementary Fig. S19**A1** and **A2**, respectively. From Supplementary Fig. S19**A1**, running time of MvDST is 7.78 ± 1.54 minutes, while that is 0.07 ± 0.004 (SCANPY), 10.63 ± 2.87 (Giotto), 6.30 ± 2.16 (BayesSpace), 0.56 ± 0.07 (SEDR), 3.57 ± 0.41 (stLearn), 1.37 ± 0.33 (SpaGCN), 0.14 ± 0.02, and 2.21 ± 0.40 (STAGATE), respectively. Supplementary Fig. S19**A2** illustrates distributions of spaces for various algorithms for DLPFC data, where space complexity of Gitto, stLearn and DeepST much higher than others. In details, space of MvDST is 2.63 ± 0.26 Gigabytes, while that is 1.48 ± 0.16 (SCANPY), 5.51 ± 0.42 (Giotto), 1.20 ± 0.09 (BayesSpace), 1.90 ± 0.11 (SEDR), 8.55 ± 1.49 (stLearn), 1.69 ± 0.08 (SpaGCN), 2.05 ± 0.05 (STAGATE), and 9.31 ± 0.70 (DeepST), respectively. From Supplementary Fig. S19**A**, it is easy to assert that Gitto and stLearn are time- and space-consuming, hampering the applications of identification of spatial domains in SRT data. And, DeepST reduces running time by sacrificing space, whereas BayesSpace reduces space complexity by sacrificing running time. SCANPY, SEDR and SpaGCN are efficient in terms of running time and space, whereas performance of these is undesirable. In other words, these algorithms improve efficiency by sacrificing accuracy of algorithms. MvDST reaches a good tradeoff between space and running time. Furthermore, it achieves the best performance on the identification of spatial domains, demonstrating that MvDST provides an

excellent alternative for current algorithms.

On the MERFISH data, we validate the efficiency of the algorithms by increasing the number of spots/cells from 1,000 to 20,000. Since SEDR, SpaGCN, and DeepST employ graph convolutional network (GCN), and DeepST is superior to them in terms of accuracy. Therefore, only SCANPY, DeepST and MvDST are selected for a comparison. Supplementary Fig. S19**B1** and **B2** describe the running time and space of algorithms with various sizes of data respectively, where MvDST reduces space complexity by sacrificing running time. These results demonstrate that MvDST reaches a good balance between time and space with the best performance of identifying spatial domains.

1.8 Accelerating MvDST with GPU

Even though we demonstrate that MvDST reaches a good balance between time and space with the best performance of identifying spatial domains, its efficiency is still undesirable since it fails to address large-scale SRT data. To solve this problem, we accelerate it with graphic processing unit (GPU), i.e., accelerating MvDST with hardware. The running time and space of MvDST with CPU and GPU for DPLDC are summarized in Supplementary Fig. S19**C1** and **C2** respectively, where MvDST (GPU) accelerates 4~5 times by sacrificing extra 50% space, demonstrating that GPU is promising for improving applicability of MvDST. The acceleration strategy is also efficient for MERFISH, where the improvement of time and space is shown in Supplementary Fig. S19**D1** and **D2**, respectively. In conclusion, MvDST reaches a good balance between time and space, and it achieves the best performance with the acceptable running time. Furthermore, GPU dramatically accelerates speed of MvDST, which is applicable for large-scale SRT data.

Supplementary Table

Table S1. Statistics of all real SRT datasets used in this study

Platform	Resolution	Distance	Tissue	Section	Spots/Cells	K_num	Enhance_ratio
**10X Visium**	55μm	100μm	Human dorsolateral prefrontal cortex (DLPFC)	151507, 151508, 151509, 151510, 151669, 151670, 151671, 151672, 151673, 151674, 151675, 151676.	4226, 4384, 4789, 4634, 3661, 3498, 4110, 4015, 3639, 3673, 3592, 3460.	18	0.2
			Human breast cancer	V1	3798		
**STARmap**	Single-cell	-	Mouse visual cortex	-	817	15	0.8
**osmFISH**	Single-cell	-	Mouse cortex	-	5328	15	0.8
**Stereo-seq**	0.22μm	subcellular	Mouse olfactory bulb	-	19527	15	0.8
**SeqFISH**	Single-cell	-	Mouse Embryogenesis	-	19451	15	0.8

Table S2. Parameter settings used in simulation experiments

**Experiment**	**Purpose**	**Ground truth cluster**	**Cluster merge probability**	**Sample size**	**Latent code dimension**	**Feature dimension**		**Noise σ**		**Dropout coefficient**	
						Trans.	Morph.	Trans.	Morph.	Trans.	Morph.
Figures 2A,B,C	Performance with Gaussian random noise of increasing variance	10	0.7	1,000	30	500	500	from 0 to 0.9 in increments of 0.1	0.1	0.5	0.1
Figures S1A, S3, S4	Performance as data quality in one modality degrades by dropouts	10	0.7	1,000	30	500	500	0.1	0.1	from 0 to 0.9 in increments of 0.1	0.1
Figures S1B, S2	Performance as the number of ground-truth subpopulations increases	6,10,15	0.7	1,000	30	500	500	0.1	0.1	0.5	0.1

Reference

[1] Bao, F., Deng, Y., Wan, S., Shen, S. Q., Wang, B., Dai, Q., ... & Wu, L. F. (2022). Integrative spatial analysis of cell morphologies and transcriptional states with MUSE. Nat Biotechnol, 40, 1200-1209.

[2] Wang Y, Liu Z, Ma X. (2025). MuCST: restoring and integrating heterogeneous morphology images and spatial transcriptomics data with contrastive learning. Genome Medicine, 17(1): 21.

[3] Wang, B., Zhu, J., Pierson, E., Ramazzotti, D., & Batzoglou, S. (2017). Visualization and analysis of single-cell RNA-seq data by kernel-based similarity learning. Nat Methods, 14, 414-416.

[4] Deng, Y., Bao, F., Dai, Q., Wu, L. F., & Altschuler, S. J. (2019). Scalable analysis of cell-type composition from single-cell transcriptomics using deep recurrent learning. Nat Methods, 16, 311-314.

Supplementary Figures

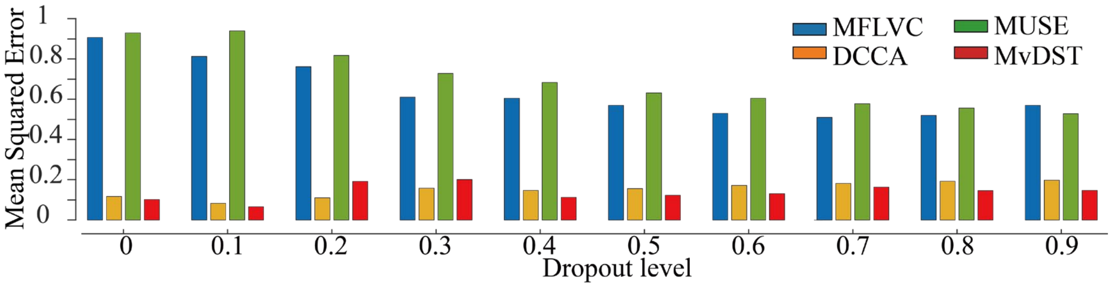

Figure S1 The Mean Squared Error between the truth clean data and denoised data obtained by various algorithms as the dropout level increases from 0 to 0.9.

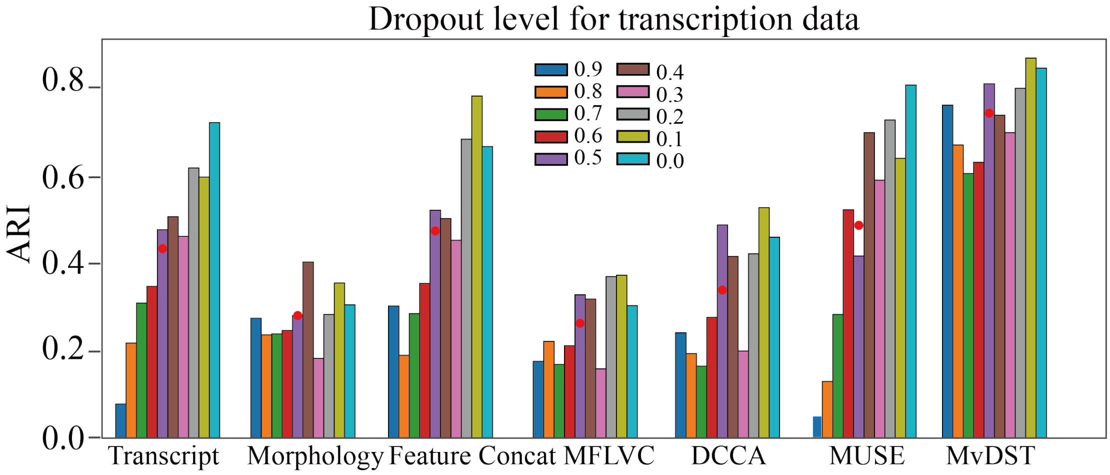

Figure S2 Performance of various algorithms on the simulated data. ARIs of identifying ground truth clusters by varying the range of dropout levels from the transcriptional modality, where the red points correspond to the average values of different dropout levels.

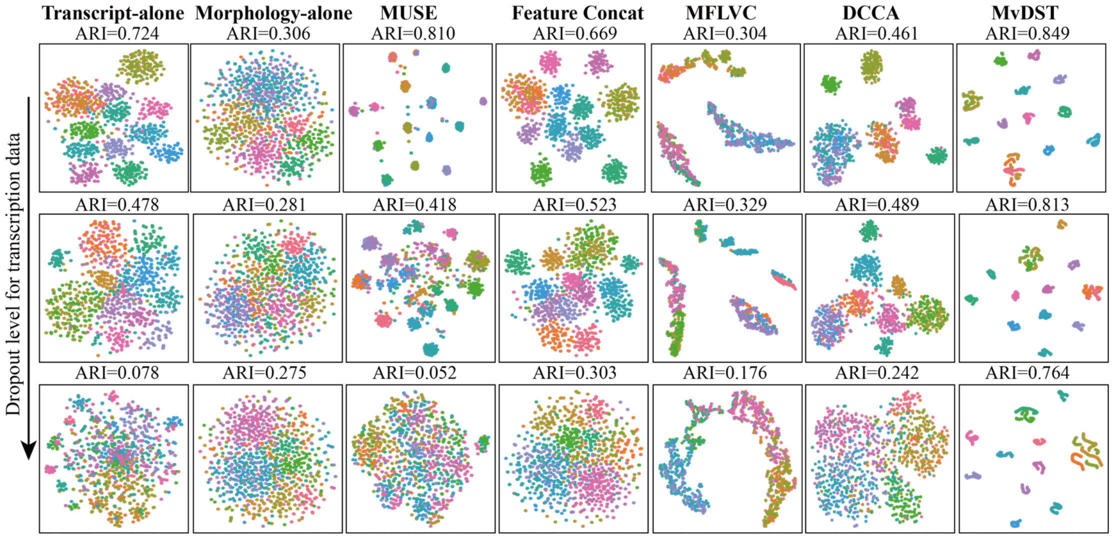

Figure S3 Performance of various algorithms on the simulated data. t-SNE visualizations of latent representations from single- and combined-modality methods with different dropout level, where ground truth subpopulation is labeled with various colors in simulation.

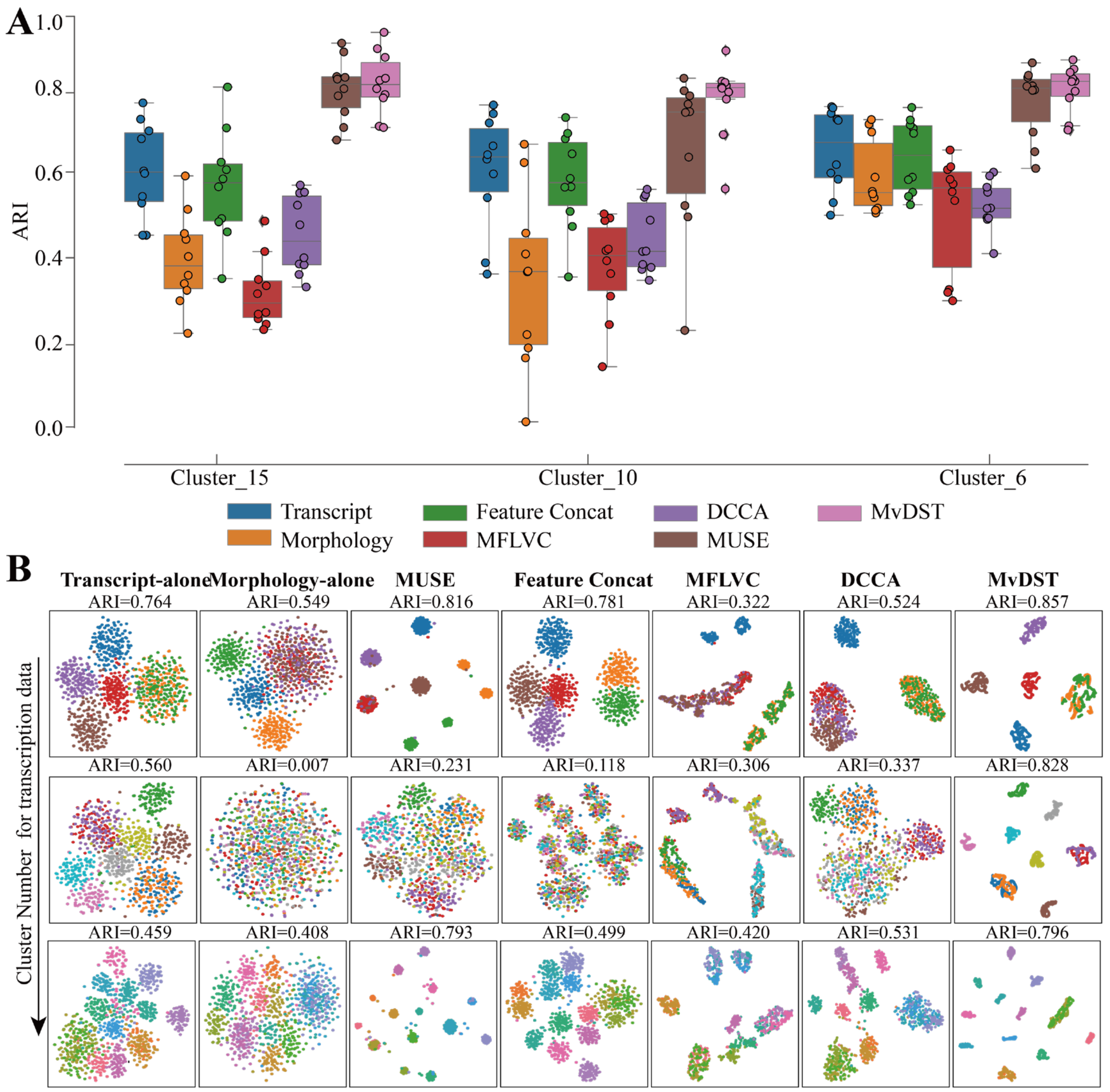

Figure S4 Performance of various algorithms on the simulated data. (**A**) ARI of identifying ground truth high-resolution subpopulations from lower-resolution single-modality subpopulations (k=15, 10 or 6), where 1,000 cells with transcriptional and morphological profiles are simulated. And, box plot is based on 10 replicates with median (center line), interquartile range (box) and data range (whiskers). (**B**) t-SNE visualizations of latent representations from single- and multi-modal methods for simulation experiments with different cluster numbers.

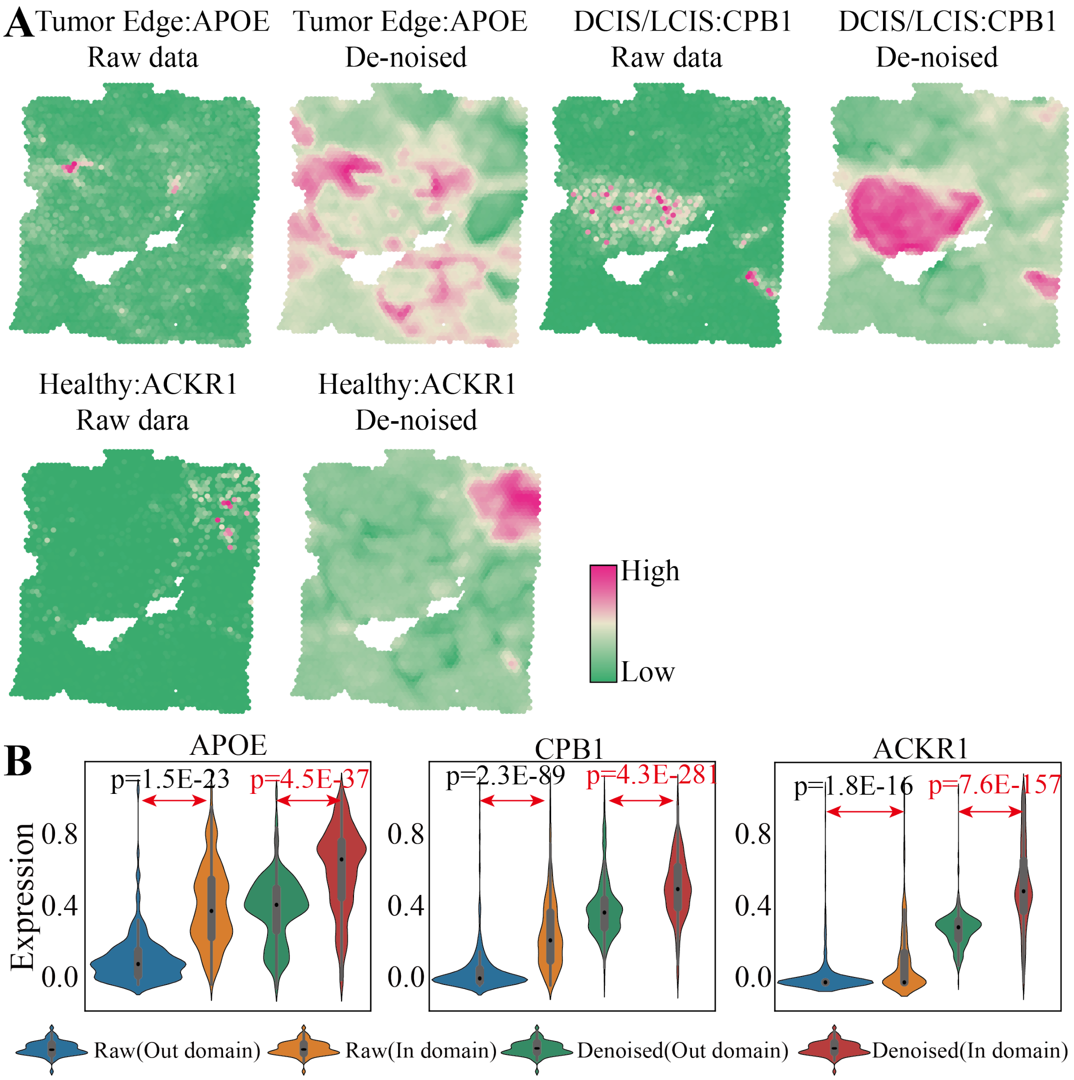

Figure S5 Performance of various algorithms in denoising cancer spatially resolved data. (**A**)Visualizations of the raw (left), reconstructed data (right) in breast cancer data. (**B**) The expression of domain-marker genes in breast cancer data, where each column corresponds to one domain (Student's t-test for significance).

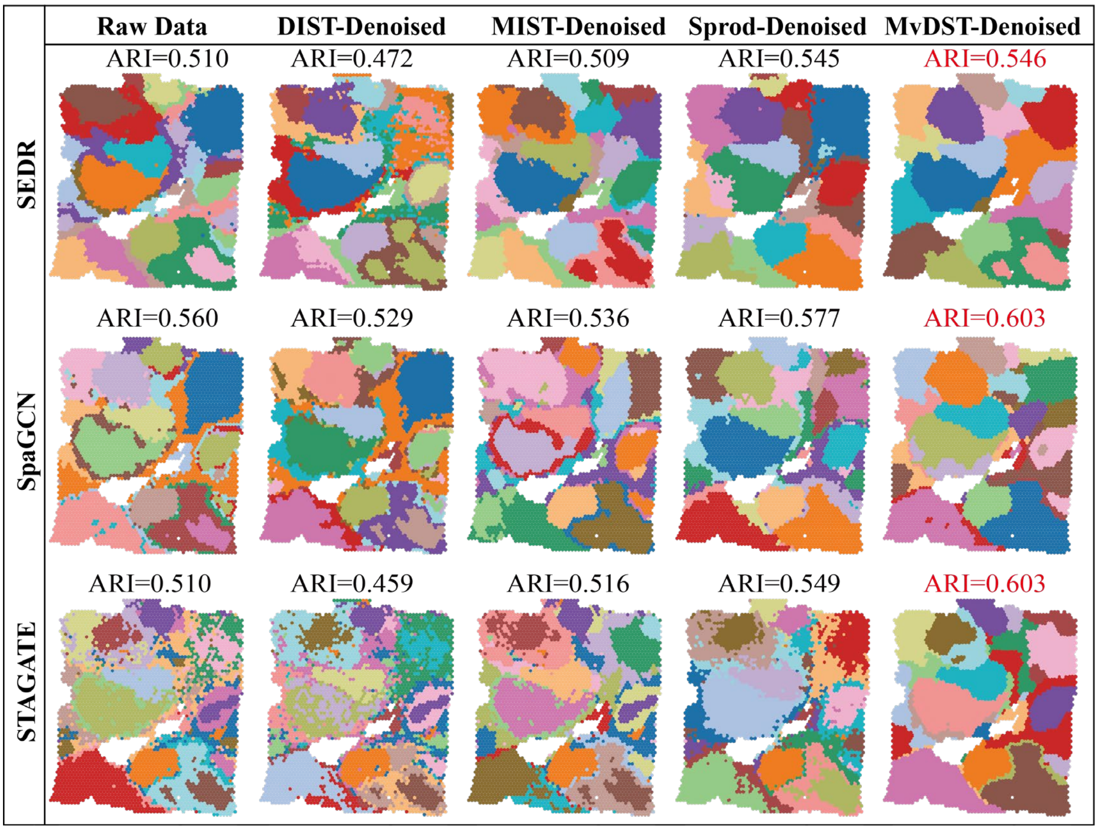

Figure S6 Performance of various algorithms in denoising cancer spatially resolved data. Visualization of spatial domains identified by various algorithms with variants of datasets, where each row denotes an algorithm, and each column corresponds visualization of spatial domains identified by various algorithms with the raw and denoised data with various approaches, such as DIST, MIST, Sprod, and MvDST, respectively.

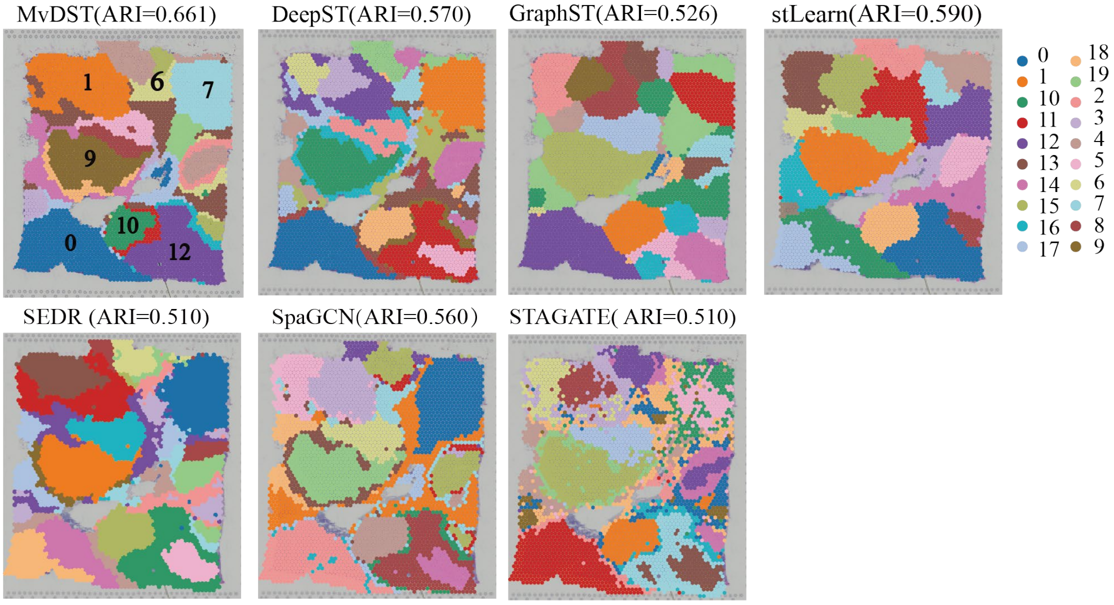

Figure S7 Performance of various algorithms for spatial domain identification for human breast spatial transcriptomics data.

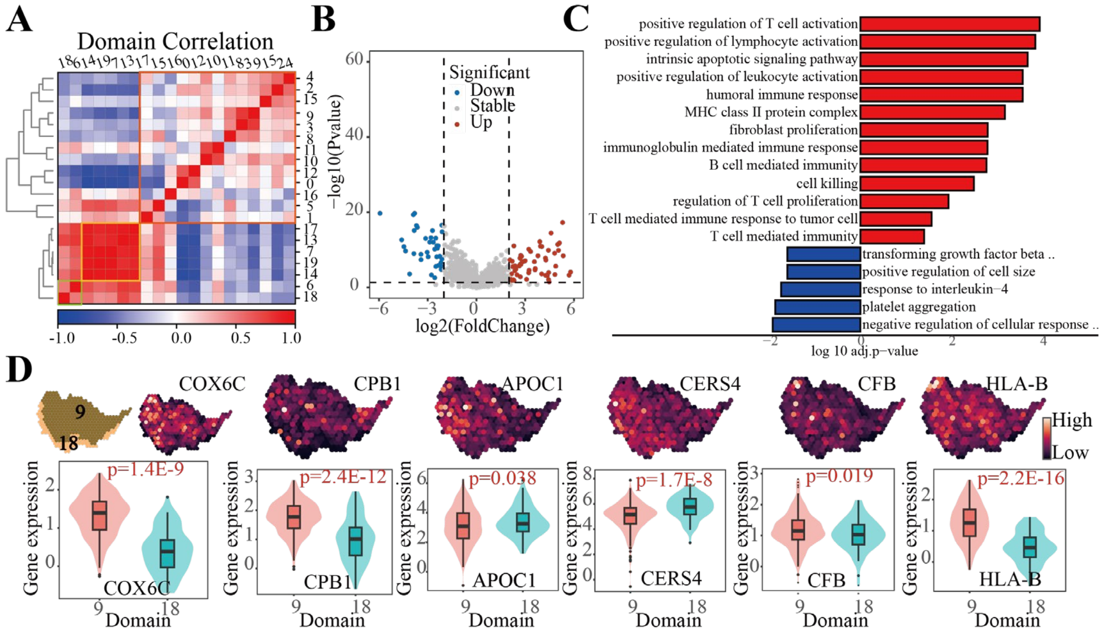

Figure S8 Performance of various algorithms for spatial domain identification for human breast spatial transcriptomics data. (**A**) Heatmap of Pearson correlation coefficient among domains. (**B**) Volcano plot of DEGs, where x-axis denotes log2(Fold Change), and y-axis represents -log10(padj-value) and (**C**) Biological functions significantly enriched by up- (red) and down-regulated (blue) DEGs, where x-axis denotes -log10(padj-value) (hypergeometric test for significance). (**D**) Visualization of expression of COX6C, CPB1, APOC1, CERS4, CFB, and HLA-B between domain 9 and 18 (top), and violin plots of expression of these two genes (bottom).

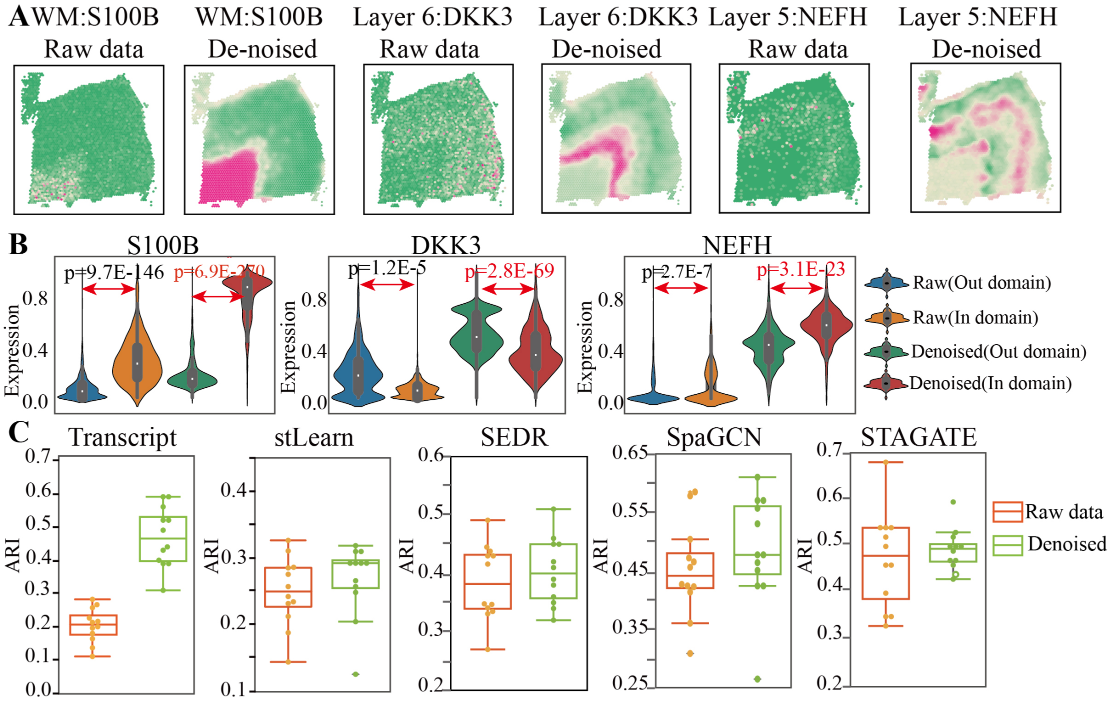

Figure S9 Performance of various algorithms in denoising normal tissues. (A) Visualizations of the original (left), reconstructed data (right) in slice 151675. (**B**) The expression of layer-marker genes in slice 151675, where each column corresponds to one layer (Student's t-test for significance). **(C)** Distributions of ARIs of various algorithms for identifying spatial domains with the original and reconstructed DLPFC data respectively, where y-axis denotes ARI and Student's t-test is for significance.

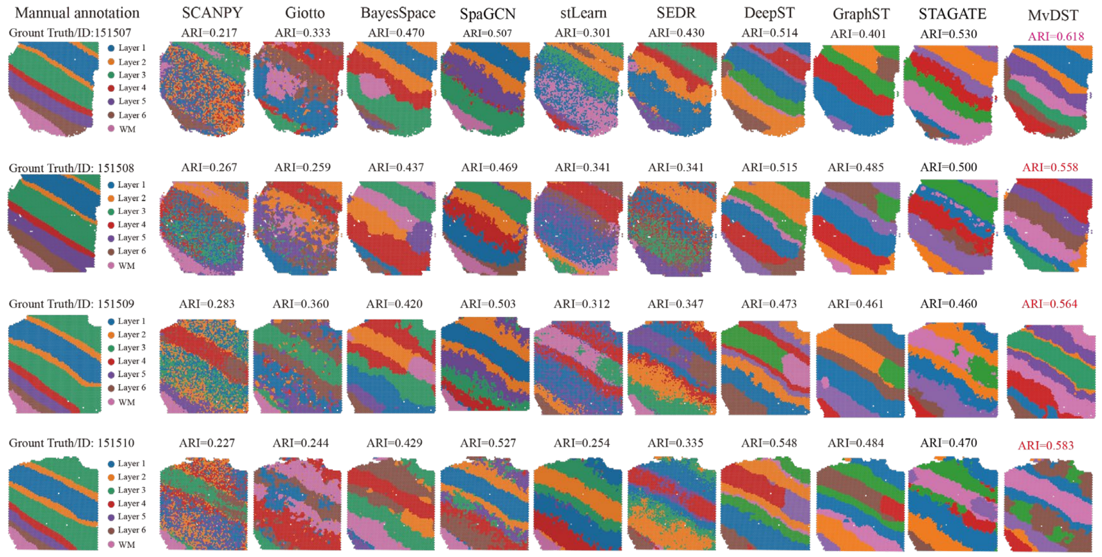

Figure S10 Performance of various algorithms for spatial domain identification on Annotated dorsolateral prefrontal cortex (DLPFC, http://spatial.libd.org/spatialLIBD) data (151507, 151508, 151509, 151510), where ground truth spots are mapped on their spatial location, divided into various cortical layers (L1-L6) and white matter (WM) layer, and each column corresponds to performance of an algorithm for various slices in terms of ARI.

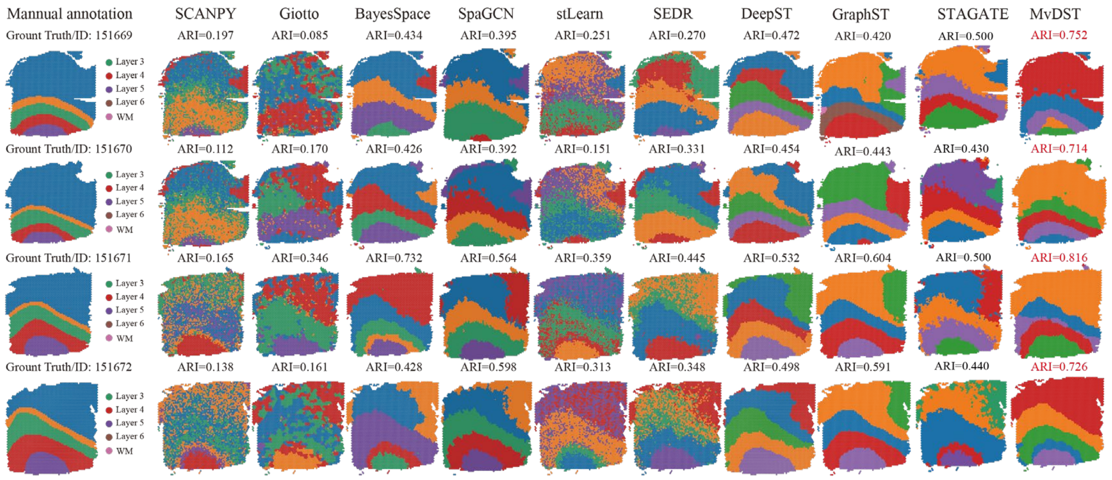

Figure S11 Performance of various algorithms for spatial domain identification on Annotated dorsolateral prefrontal cortex (DLPFC, http://spatial.libd.org/spatialLIBD) data (151669, 151670, 151671, 151672), where ground truth spots are mapped on their spatial location, divided into various cortical layers (L3-L6) and white matter (WM) layer, and each column corresponds to

performance of an algorithm for various slices in terms of ARI.

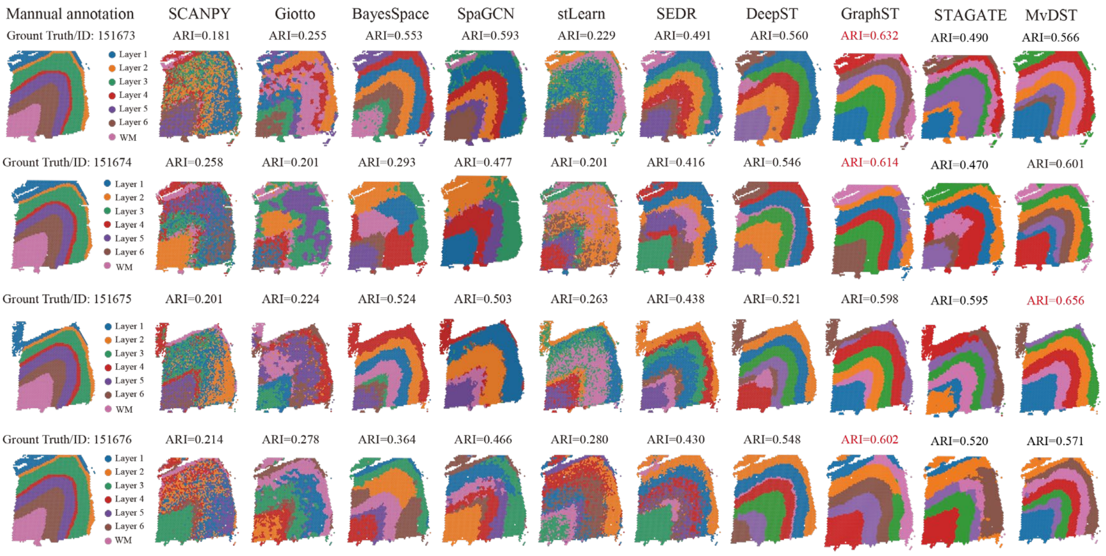

Figure S12 Performance of various algorithms for spatial domain identification on Annotated dorsolateral prefrontal cortex (DLPFC, http://spatial.libd.org/spatialLIBD) data (151673, 151674, 151675, 151676), where ground truth spots are mapped on their spatial location, divided into various cortical layers (L1-L6) and white matter (WM) layer, and each column corresponds to performance of an algorithm for various slices in terms of ARI.

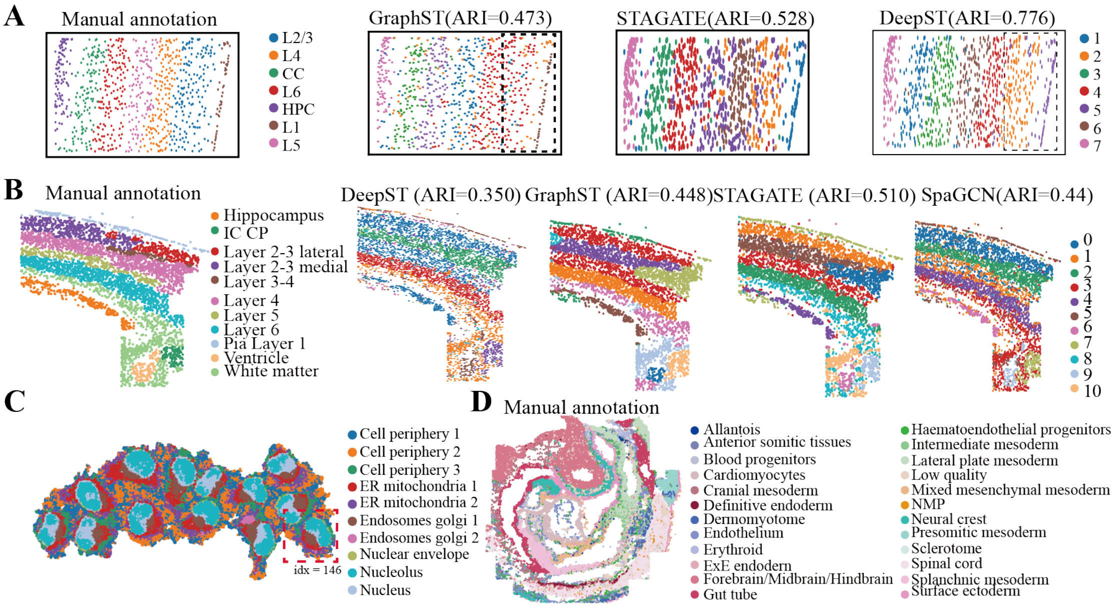

Figure S13 MvDST handles data from various platforms. (**A**) Manual annotation of STARmap data (left), and spatial domain identified by GraphST, STAGATE, and DeepST. (**B**) Manual annotation of osmFISH data (left), and spatial domains identified by DeepST, GraphST, STAGATE, and SpaGCN. (**C**) Visualization of subcellular molecular profiles using the full 4i (iterative indirect immunofluorescence imaging). (**D**) Annotation of SeqFISH-profiled mouse embryo tissue sections, which was obtained from the original publication, and Spatial domains identified by MvDST using the SeqFISH data with a fixed number of clusters (k=24) as a

clustering parameter.

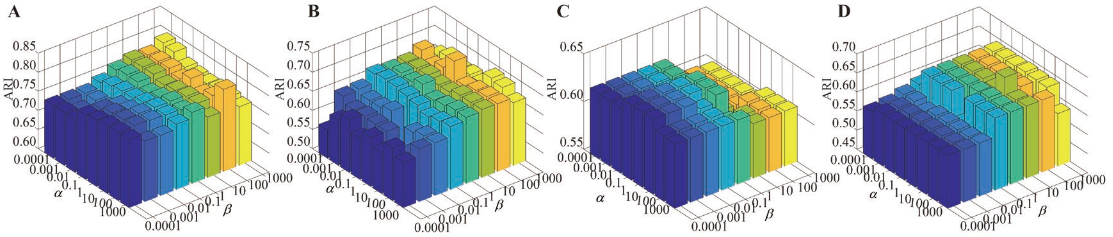

Figure S14 Parameter analysis of MvDST on various datasets, **(A)** Balanced simulated dataset, **(B)** Unbalanced simulated dataset, **(C)** the DLPFC dataset, and **(D)** human breast cancer dataset.

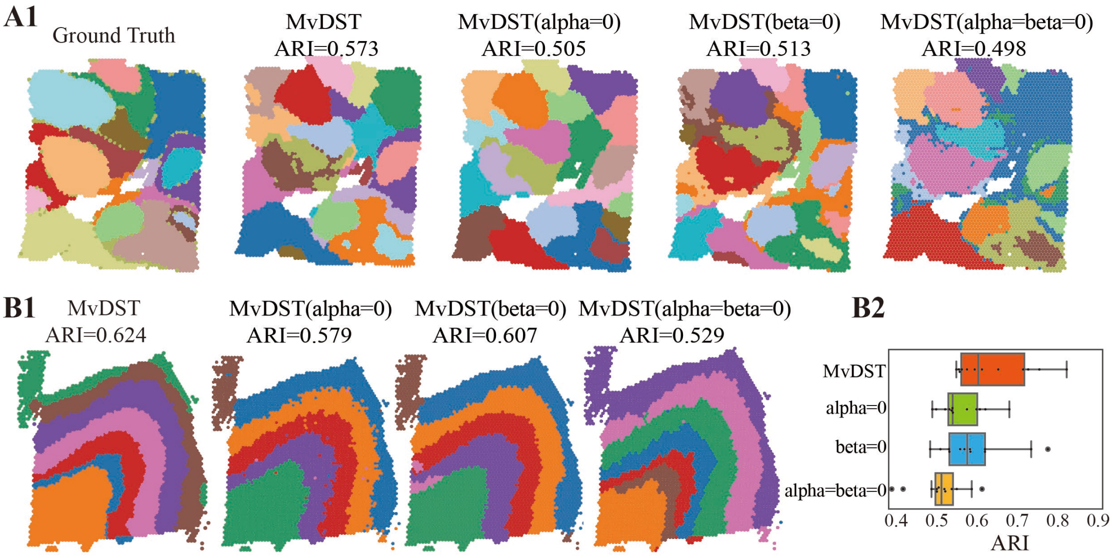

Figure S15 The ablation analysis of MvDST in terms of performance among MvDST(α=0), MvDST(β=0), and MvDST(α=0, β=0) on different datasets. **(A1)** ARI of methods on the breast cancer dataset, **(B1)** ARI of methods on the slice 151675 of DLPFC dataset, and **(B2)** ARIs of various algorithms on all 12 slices of DLPFC dataset.

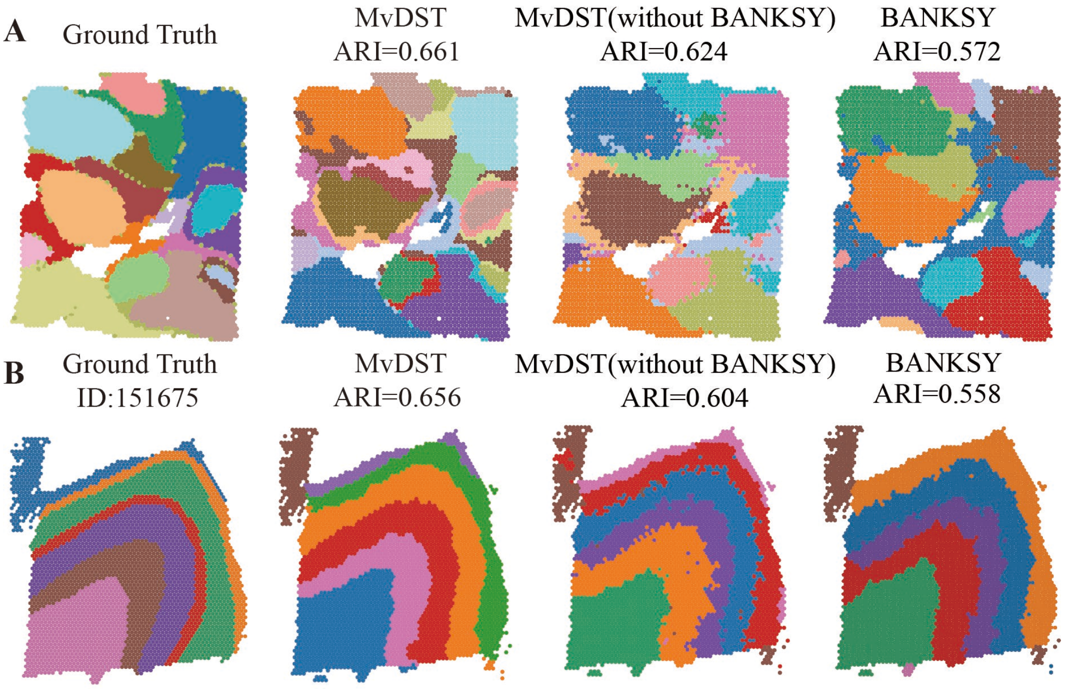

Figure S16 Ablation analysis of MvDST with various datasets. (A) Visualization of spatial domains identified by MvDST (without BANSKY), MvDST and BANKSY for breast cancer dataset, **(B)** Visualization of spatial domains identified by MvDST (without BANSKY), MvDST and BANKSY on the slice 151675 of DLPFC dataset.

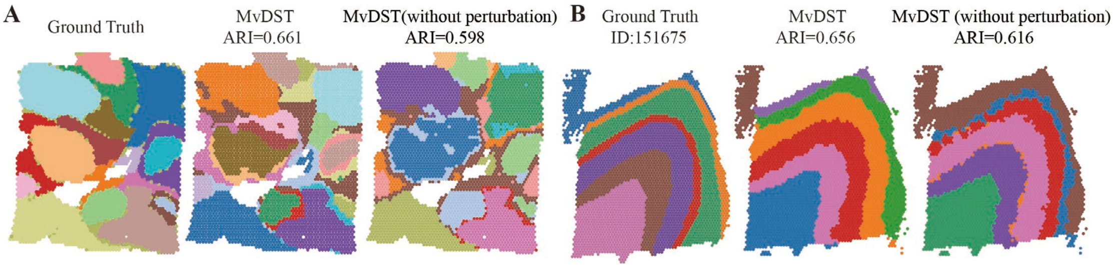

Figure S17 Ablation analysis of MvDST in terms of performance without perturbation across different datasets. (A1) Spatial domains identified by applying K-means clustering to denoised data generated by different algorithms on the human breast cancer dataset, and **(A2)** Spatial domains identified by applying K-means clustering to denoised data generated by different algorithms on slice 151675 of the DLPFC dataset.

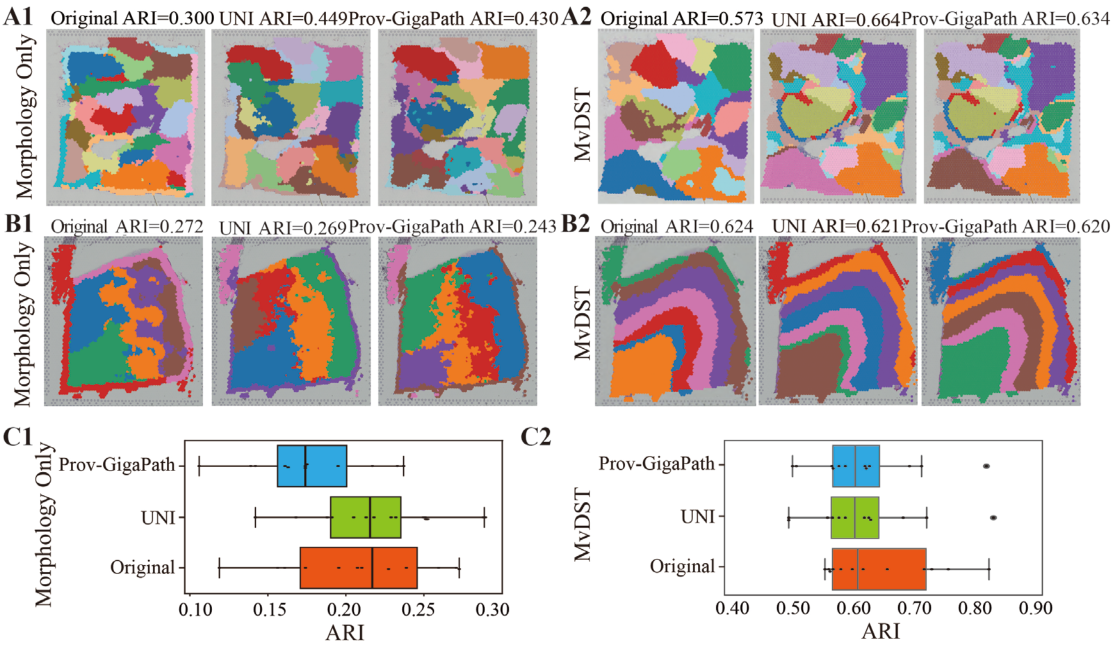

Figure S18 Performance of biological image-specific models and MvDST on various datasets. (A) the human breast cancer dataset, **(B)** slice 151675 of DLPFC dataset, and **(C)** all 12 slices of DLPFC, where y-axis denotes ARI, and the center line, box limits and whiskers denote the median, upper and lower quartiles, and 1.5 × interquartile range, respectively.

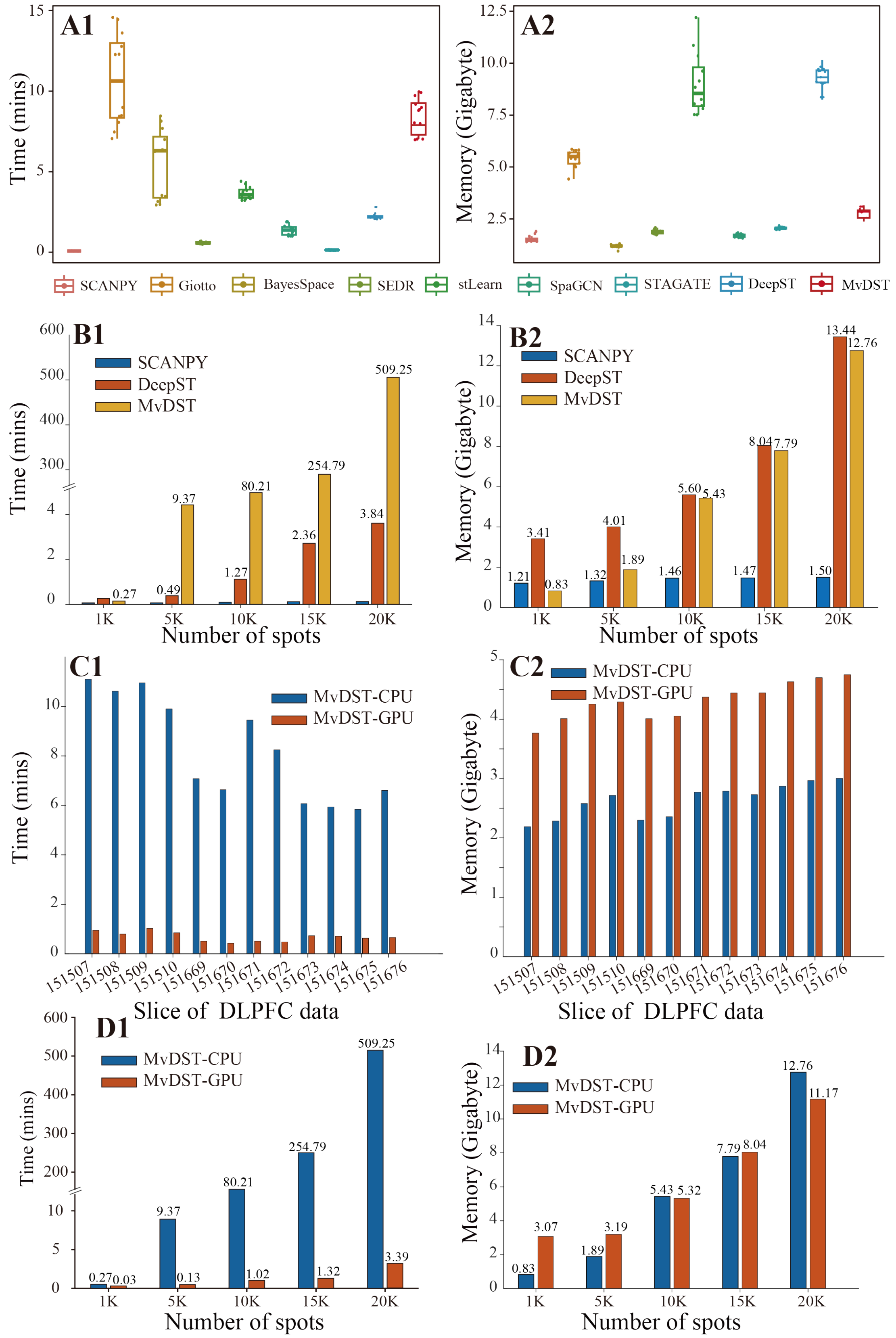

Figure S19 Running time and space of algorithms for different spatial transcriptomics data. **(A1/A2)** Distributions of running time (minutes) and space (Gigabyte) of algorithms on the DLPFC data. **(B1/B2)** Running time and space of algorithms on MERFISH data with various sizes.

(C1/C2) Running time and space of MvDST with and without acceleration on the DLPFC data.

(D1/D2) Running time and space of MvDST with and without acceleration on the MERFISH data.
